# SNX10-mediated degradation of LAMP2A by NSAIDs inhibits chaperone-mediated autophagy and induces hepatic lipid accumulation

**DOI:** 10.7150/thno.70692

**Published:** 2022-02-21

**Authors:** Wonseok Lee, Hyun Young Kim, You-Jin Choi, Seung-Hwan Jung, Yoon Ah Nam, Yunfan Zhang, Sung Ho Yun, Tong-Shin Chang, Byung-Hoon Lee

**Affiliations:** College of Pharmacy and Research Institute of Pharmaceutical Sciences, Seoul National University, Seoul, Republic of Korea.

**Keywords:** Chaperone-mediated autophagy, Diclofenac, NSAIDs, Perilipin 2, Sorting nexin 10

## Abstract

**Rationale:** While some non-steroidal anti-inflammatory drugs (NSAIDs) are reported to induce hepatic steatosis, the molecular mechanisms are poorly understood. This study presented the mechanism by which NSAIDs induce hepatic lipid accumulation.

**Methods:** Mouse primary hepatocytes and HepG2 cells were used to examine the underlying mechanism of NSAID-induced hepatic steatosis. Lipid accumulation was measured using Nile-red assay and BODIPY 493/503. The activity of chaperone-mediated autophagy (CMA) was determined by western blotting, qRT-PCR, and confocal imaging. The effect of NSAID on CMA inhibition was evaluated *in vivo* using diclofenac and CMA activator (AR7) administered mice.

**Results:** All tested NSAIDs in this study accumulated neutral lipids in hepatocytes, diclofenac having demonstrated the most potency in that regard. Diclofenac-induced lipid accumulation was confirmed in both mouse primary hepatocytes and the liver of mice. NSAIDs inhibited CMA, as reflected by the decreased expression of lysosome-associated membrane glycoprotein 2 isoform A (LAMP2A) protein, the increased expression of CMA substrate proteins such as PLIN2, and the decreased activity of photoactivatable KFERQ-PAmCherry reporter. Reactivation of CMA by treatment with AR7 or overexpression of LAMP2A inhibited diclofenac-induced lipid accumulation and hepatotoxicity. Upregulation of sorting nexin 10 (SNX10) via the CHOP-dependent endoplasmic reticulum stress response and thus maturation of cathepsin A (CTSA) was shown to be responsible for the lysosomal degradation of LAMP2A by diclofenac.

**Conclusion:** We demonstrated that NSAIDs induced SNX10- and CTSA-dependent degradation of LAMP2A, thereby leading to the suppression of CMA. In turn, impaired CMA failed to degrade PLIN2 and disrupted cellular lipid homeostasis, thus leading to NSAID-induced steatosis and hepatotoxicity.

## Introduction

Lipid droplets (LDs) are dynamic cytoplasmic organelles serving an essential function as lipid reservoirs and providing substrates for energy metabolism. LDs consist of a neutral lipid core encircled by a phospholipid monolayer and a family of coat proteins such as perilipins (PLINs). Synthesis and degradation of LDs are controlled by diverse cellular signaling in response to the energy and metabolic balance. Degradation of LDs occurs either through enzymatic lipolysis or autophagy: lipolysis is characterized by phosphorylation and proteasomal degradation of PLIN1 and subsequent activation of adipose triglyceride lipase 1; in autophagy, a small portion of LDs are selectively sequestered in autophagosomes and delivered to lysosomes for degradation 2. A recent study demonstrated that selective removal of the LD surface proteins, PLIN2 and PLIN3, by chaperone-medicated autophagy (CMA) is the initial step facilitating the degradation of the lipid component of the LD core by lipolysis or lipophagy 3. Therefore, PLINs are believed to play a role as gatekeepers in LD mobilization. In a CMA-deficient model system, the degradation of the coat proteins was inhibited and the association of autophagy proteins and lysosome-associated membrane glycoprotein 1 (LAMP1) with LDs was decreased, resulting in LD accumulation and steatosis 4.

PLIN2 is one of the representative CMA substrate proteins associated with lipid metabolism. Chaperone heat shock cognate 71 kDa protein (HSC70) recognizes and binds to the KFERQ-like motif of PLIN2 and transports it to the LAMP2A for degradation 5. The selective degradation of cytosolic proteins by CMA depends, at least in part, on the availability of LAMP2A receptor at the lysosomal membrane. LAMP2A knockout results in a decreased CMA activity, which leads to increased PLIN2 expression, decreased lipid oxidation and subsequent LD accumulation 4, 6. LAMP2A being the rate-limiting factor of CMA, its expression and/or stabilization of the protein is crucial to that process. Several transcription factors are reported to regulate LAMP2A expression. For example, a redox-sensitive transcription factor, namely nuclear factor erythroid 2-related factor 2 (NRF2), controls the basal and inducible expression of LAMP2A and CMA activity 7. Sorting nexin 10 (SNX10) facilitates the trafficking of cathepsin A (CTSA) into lysosomes where it is processed into its active form. Active CTSA interacts with and stimulates the degradation of LAMP2A on the lysosomal membrane. Cells defective in CTSA show reduced rate of LAMP2A degradation, higher levels of LAMP2A, and higher rates of CMA 8. Interestingly, SNX10 knockout mice exhibited significantly ameliorated ethanol-induced hepatic steatosis and injury via inhibition of CTSA maturation, LAMA2A stabilization and, thus, CMA activation 9.

Non-steroidal anti-inflammatory drugs (NSAIDs) are among the most widely used analgesic and anti-inflammatory agents worldwide. Although gastrointestinal adverse events are most common, hepatic injury is another consequence of NSAIDs use. Some NSAIDs, in fact, have been withdrawn from the market due to fatal cardiovascular and hepatic toxicity 10. Mitochondrial damage and the generation of reactive oxygen species (ROS) are the key initiating events associated with NSAID-induced hepatotoxicity 11, 12. Recently, we reported that ROS-mediated lysosomal dysfunction and suppression of autophagy fail to maintain mitochondrial integrity and aggravate cellular oxidative stress, leading to NSAID-induced hepatotoxicity 13. Some NSAIDs have been reported to induce hepatic steatosis, including some fatal cases, by inhibiting mitochondrial β-oxidation of fatty acids irrespective of their cyclooxygenase (COX)-inhibiting properties 14-16. However, little is known about the effects of NSAIDs on lipid metabolism and whether CMA is involved in this process. In the present study, we investigated the role of CMA in NSAID-induced hepatic lipid accumulation. We found that NSAIDs, including diclofenac, increase lysosomal degradation of LAMP2A and inhibit CMA activity. LAMP2A degradation is caused by upregulation of SNX10 and maturation of CTSA. Impairment of CMA-mediated PLIN2 degradation disrupts cellular lipid homeostasis, which leads to diclofenac-induced steatosis and hepatotoxicity.

## Material and Methods

### Cell Culture

Mouse primary hepatocytes were isolated from specific pathogen-free male C57BL/6J mice (23-25 g) by perfusion of the liver using collagenase type Ⅳ and maintained in Williams' medium E (Sigma, W4128) as described previously 17. HepG2 (human hepatoma cell line) cells and SN4741 (murine nigral dopaminergic cell line) cells were maintained in Dulbecco's modified Eagle's medium (DMEM; Hyclone, SH30021.01) supplemented with 10% fetal bovine serum (FBS; Gibco, 16000044) and 1% antibiotic-antimycotics (Gibco, 15250062). AML12 (murine hepatocyte cell line) cells were maintained in DMEM/F12 medium (Gibco, 11320033) containing 10% FBS, 1% insulin-transferrin-selenium (Gibco, 41400-045), 40 ng/ml dexamethasone (Sigma, D4902), and 1% antibiotic-antimycotics. HepG2 and AML12 cells were incubated at 37 °C, and SN4741 cells were incubated at 33 °C with air containing 5% CO_2_. HepG2 and AML12 cells were purchased form American Type Culture Collection (ATCC) and SN4741 was kindly gifted from Dr. Yong-Keun Jung (Seoul National University, Korea).

### Antibodies and Chemicals

Antibodies against ATF6 (65880) Bip (3183), CHOP (2895), COX2 (12282), GAPDH (2118), LC3B (2775) and PK (7067) were purchased from Cell Signaling Technology. Antibodies against PLIN2 (NB110-40877) and SNX10 (NBP1-79562) were purchased from Novus Biologicals. Antibodies against LAMP2A (ab18528) and COX1 (ab109025) were purchased from Abcam. Antibodies against β-actin (sc-47778) and IRE1α (sc-390960) were purchased from Santa Cruz Biotechnology. Antibodies against CTSA (Origene; TA332731) LAMP1 (Developmental Studies Hybridoma Bank; 1D4B) were obtained as indicated. BODIPY493/503 (D3922) and DAPI solution (62248) were purchased from Thermo Fisher Scientific. BAPTA-AM (A1076), diclofenac sodium (D6899), Mito-Tempo (SML0737), oleate (O1008), leupeptin (L2884), rapamycin (553210), clioquinol (ClioQ; 233165), Nile Red (72485), 4-phenylbutyrate (SML0309), taurodeoxycholic acid sodium salt (T0266) and tunicamycin (T7765) were purchased from Sigma Aldrich.

### Nile Red Assay

Intracellular lipid accumulation was quantified using Nile Red, a fluorescent dye that binds to neutral lipids. Cells were seeded in 96 well plates and incubated with chemicals for 24 h. On the following day, cells were fixed with 4% paraformaldehyde and stained with 1 μg/ml Nile Red solution. The fluorescence was measured with a microplate fluorescence reader (Molecular Devices) at excitation and emission wave lengths of 488 nm and 580 nm, respectively.

### Determination of TG

Total lipids were extracted from liver homogenates prepared from 100 mg of livers and primary hepatocytes using chloroform/methanol mixture (2:1, v/v). TG concentration was determined enzymatically using a commercially available enzymatic kit (TR0100) from Sigma-Aldrich according to the manufacturer's protocol.

### Animal experiments

Male C57BL/6 mice (aged 7 weeks) were purchased from Jackson Laboratory via Orient Bio. Only male mice were used for our animal experiment to exclude any sex-related variability to identify basic biological effects. Mice were housed in an air-conditioned room (24 °C) with a 12 h light/dark cycle, and allowed to free access to water and food for 1 week. After 1-week adaption period, the animals were randomly divided to the following 4 groups (6 mice per group) - Group 1, vehicle; Group 2, diclofenac; Group 3, AR7; Group 4, diclofenac + AR7. The vehicle groups were administered intraperitoneally with 0.5% DMSO in saline. Diclofenac groups were administered diclofenac (100 mg/kg, i.p) twice every 12 h. AR7 groups were administered AR7 (10 mg/kg, i.p) 1 h before diclofenac administration. Next day, mice were anesthetized with Zoletil 50 (Virbac) and sacrificed. The serum levels of alanine transaminase, aspartate aminotransferase, TG were measured using an Automated Chemistry Analyzer (Prestige 24I; Tokyo Boeki Medical System, Tokyo, Japan) according to the manufacturer's protocol.

All animal experiments were carried out in accordance with animal experiment guidelines with the approval of the Institutional Animal Care and Use Committee of Seoul National University (approval number: SNU-200615-2-1).

### Histological analysis

Liver tissue specimens were fixed in 4% paraformaldehyde, embedded in paraffin blocks, cut into 5-µm-thick sections and stained with hematoxylin and eosin (H&E). For Oil Red-O staining, frozen liver tissues were cut into 7-µm-thick sections and mounted to microscope slides. Sections were reacted with Oil Red-O solution buffer and counterstained with Harris hematoxylin as described previously 18.

### Western Blot analysis

Cells and liver homogenates were lysed in lysis buffer containing protease inhibitor cocktail (Roche) as described previously 13. Protein bands were detected with Amersham ECL Prime Detection Reagent (GE Healthcare Life Sciences, RPN2232). Quantitative densitometry analysis was performed using Quantity One software (Bio-Rad).

### RNA preparation and qRT-PCR analysis

mRNA was extracted with the Easy-Blue Total RNA extraction kit (iNtRON) and cDNA was prepared with QuantiTect Reverse Transcription Kit (QIAGEN). cDNA was amplified by qRT-PCR using iTaq Universal SYBR Green Supermix kit (Bio-Rad). The sequences of primers used for qRT-PCR are listed in Table S1.

### Transient transfections

For transfection, HepG2 and mouse primary hepatocytes were seeded in 6 well plates 1 day before the transfection. The siRNA duplexes targeting human ATF6 (Bioneer), mouse Cathepsin A (Bioneer), human CHOP (Bioneer), human IRE1α (Bioneer), mouse SNX10 (Santacruz) or control siRNA were transfected using Lipofectamine RNAiMAX (Invitrogen, 13778075) according to the manufacturer's protocol. Mouse LAMP2A overexpression vector (Origene, RC221216), human CHOP overexpression vector (Origene, RC201301) were transfected using Lipofectamine 2000 (Invitrogen, 11668027).

### Immunofluorescence analysis and confocal microscopy

For immunofluorescence microscopy, cells were rinsed with PBS and fixed with 4% paraformaldehyde and then blocked with 1% horse serum (Gibco, 16050122) in PBS. Samples were incubated with appropriate primary antibodies at 4 °C overnight and followed by incubation with secondary Alexa-Flour 488, 596 dye-labeled antibodies. The nuclei were stained with 1 μg/ml DAPI in PBS for 10 min. For BODIPY staining, cells were loaded with 10 μg/ml BODIPY 493/503 in PBS for 20 min. Coverslips were mounted using ProLong Gold Antifade Reagent (Life Technologies, P36930) and images were captured with TCS SP8 confocal laser scanning microscope (Leica Microsystems). The confocal images were acquired using identical exposure settings on the same day in each experiment replicate. 5 images were randomly selected and quantified using Image J software.

### Measurement of intracellular Ca^2+^ levels

HepG2 cells seeded in 48-well plate were incubated with Fluo-3 AM (2 µM) for 30 min and observed using the IncuCyte S3 live cell imaging system (Essen Bioscience).

### Measurement of CMA activity using PA-mCherry-KFERQ reporter

pSIN-PAmCherry-KFERQ-NE was obtained from Addgene (plasmid # 102365). HepG2 cells seeded in 12-well plates with microscope cover glasses were infected with pSIN-PAmCherry-KFERQ-NE lentiviral particle as described previously 19. Cells were photoactivated with a 405 nm light-emitting diode LED for 5 min with the intensity of 3.5 mA. After 16 h, cells were fixed with 4% paraformaldehyde and analyzed using confocal microscopy. Quantification was performed by counting the red puncta.

### Lysosomal fractionation

Lysosomal fractions were prepared from 100 mg of livers and cultured cells using lysosome enrichment kit (Thermo Fisher Scientific, 89839) according to the manufacturer's protocol.

### Statistical analysis

The statistical analysis of the data was performed using GraphPad Prism 7 (GraphPad Software). Experimental data were presented as mean ± S.D. *in vivo* and *in vitro* experiments. Data were subjected to student's t-test or one-way ANOVA analysis or two-way ANOVA followed by Turkey's multiple comparison test. P values < 0.05 were considered statistically significant.

## Results

### NSAIDs induce hepatic lipid accumulation *in vitro* and *in vivo*

**Figure 1:**
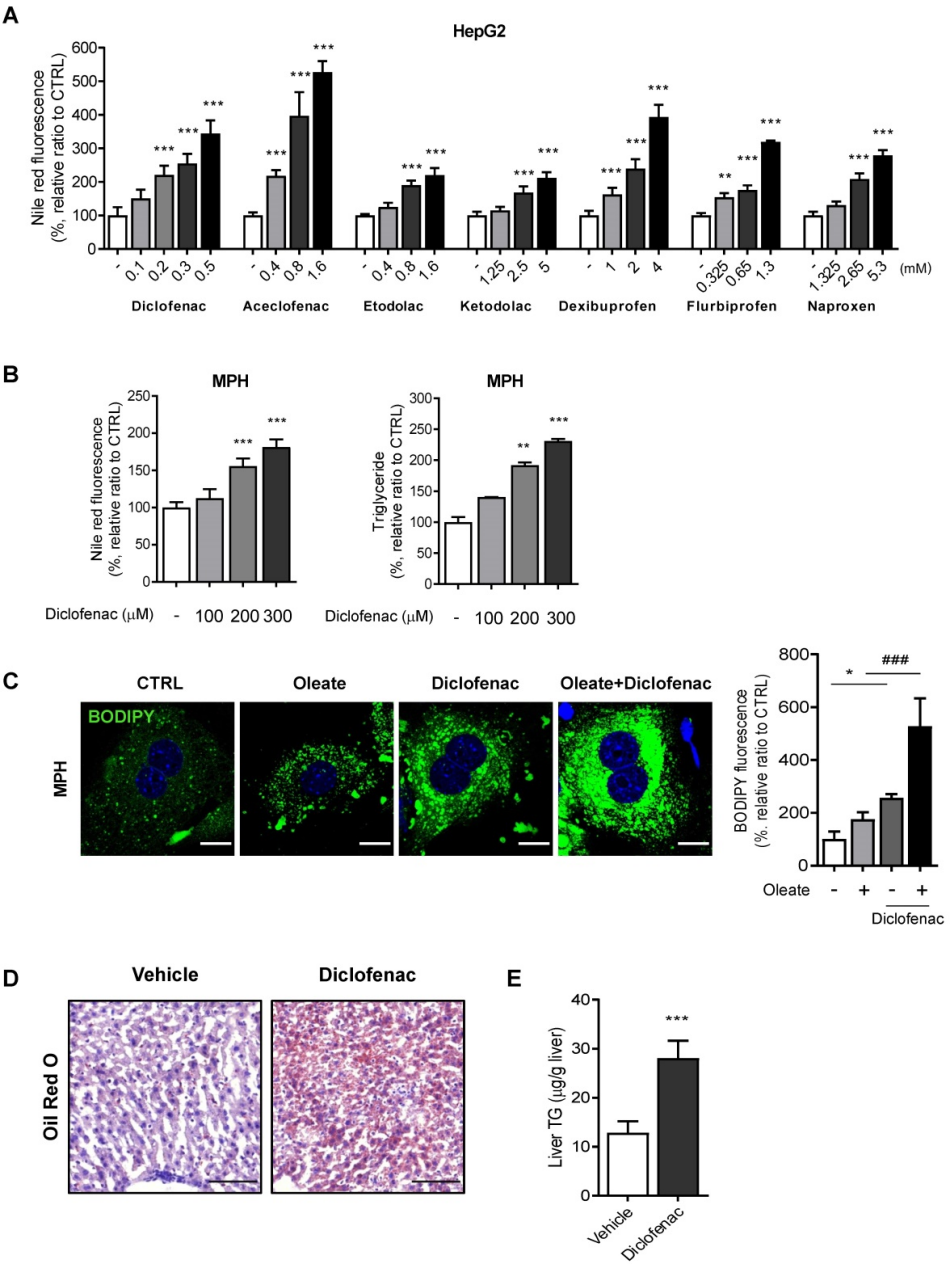
** NSAIDs induce hepatic lipid accumulation *in vitro* and *in vivo*. (A)** HepG2 cells were treated with NSAIDs for 24 h, and intracellular neutral lipid levels were quantified using Nile-red dye; the excitation and emission wavelengths were 486 and 528 nm, respectively. **(B)** MPH were treated with diclofenac at the indicated concentration for 24 h, and intracellular neutral lipid levels were quantified using Nile-red dye (left panel). Intracellular triglyceride (TG) concentrations were quantified using an enzymatic kit (right panel). **(C)** MPH were treated with diclofenac (300 µM) and/or oleate (100 µM) followed by staining with BODIPY 493/503 (green). Nuclei were stained with DAPI (blue). Representative fluorescent images and quantitative data are shown (Scale bars: 20 µm). **(D, E)** C57BL/6 mice were intraperitoneally injected with diclofenac (100 mg/kg) and sacrificed after 24 h (n = 6). (D) Representative Oil Red O-stained liver sections demonstrating neutral lipids in the liver (Scale bars: 100 µm). (E) TG concentrations in the liver of mice were quantified using the enzymatic kit. Data are presented as mean ± SD of at least 3 independent experiments, as analyzed by one-way ANOVA followed by Tukey's test. *p < 0.05, **p < 0.01, and ***p < 0.001, relative to the control group. ###p < 0.001, relative to the oleate-treated group.

We first evaluated whether NSAIDs induce neutral lipid accumulation in hepatocytes* in vitro* and *in vivo*. Based on our previous paper, we selected 7 NSAIDs, 4 from acetic acid and 3 from propionic acid derivatives 13. To exclude artifacts from the drugs' differing cytotoxicity, we set the highest concentration in all cases to half of the IC_50_ determined by an MTT assay (data not shown). All of the tested NSAIDs increased the intensity of Nile red fluorescence in HepG2 cells in a concentration-dependent manner, diclofenac being the most potent (Fig. 1A). We therefore selected diclofenac as the model compound in order to investigate the underlying mechanism of lipid accumulation. Diclofenac-induced lipid accumulation was confirmed also in mouse primary hepatocytes (MPH) by increased Nile red fluorescence and triglyceride level (Fig. 1B). MPH accumulated more and larger LDs visible with BODIPY493/503, under both the basal and oleate-treated conditions. The lipid-accumulating effects of diclofenac and oleate were synergistic (Fig. 1C). Similar results were obtained in AML12 murine normal hepatocytes and HepG2 cells (Fig. S1A-C). The intraperitoneal administration of 100 mg/kg diclofenac to mice increased the hepatic lipid accumulation as determined by Oil red O staining and liver TG level (Fig. 1D-E). These data indicate that diclofenac and other structurally related NSAIDs induce hepatic lipid accumulation both *in vitro* and *in vivo*.

### NSAIDs increase abundance of CMA substrate in hepatocytes

**Figure 2:**
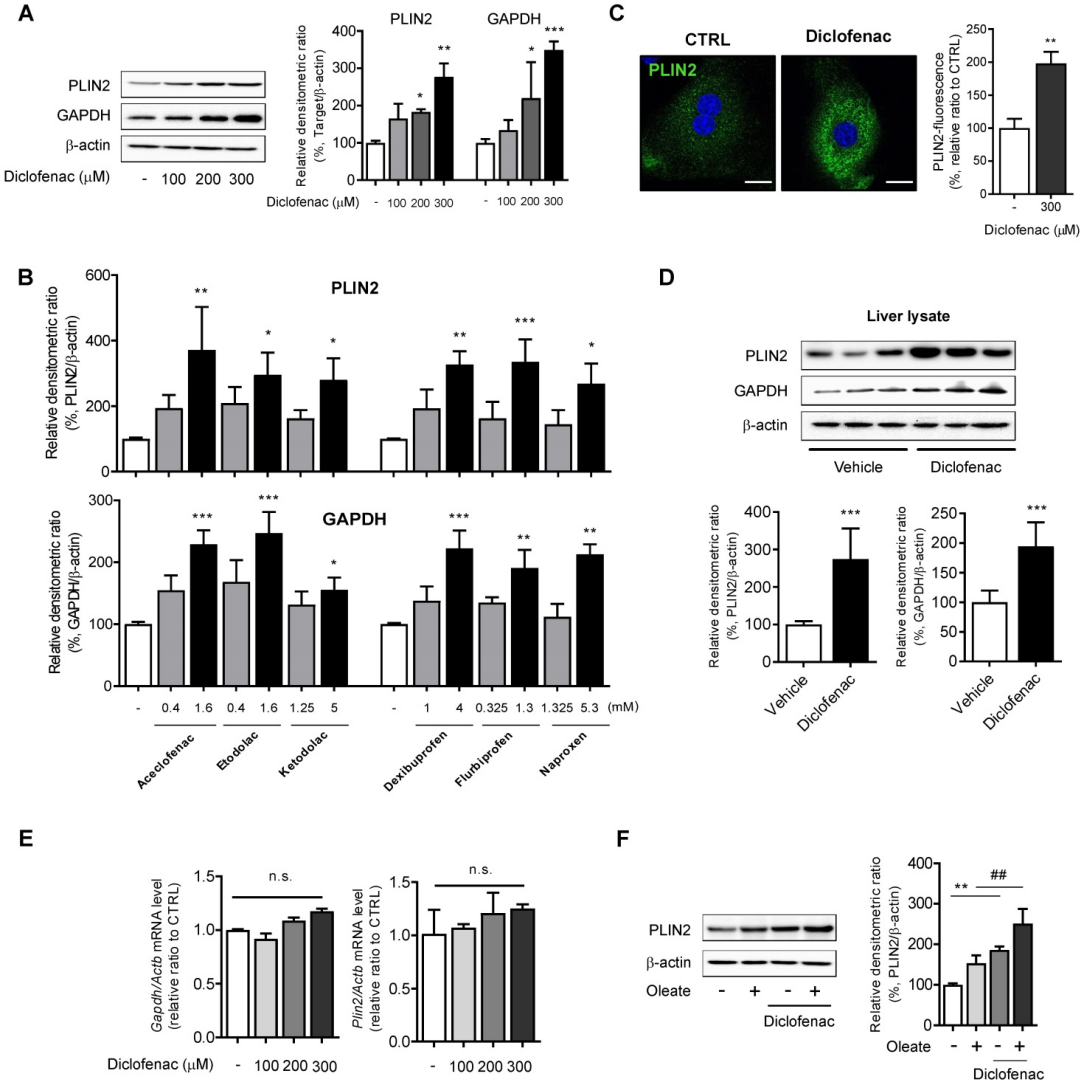
** NSAIDs accumulate CMA substrates *in vitro* and *in vivo*. (A)** Western blot analysis of PLIN2 and GAPDH in MPH, treated with the indicated concentrations of diclofenac for 24 h. The right panel shows the densitometric quantification of the PLIN2 and GAPDH levels. **(B)** HepG2 cells were treated with the indicated concentration of NSAIDs for 24 h, and the levels of PLIN2 and GAPDH were analyzed by western blotting. **(C)** MPH were treated with diclofenac (300 µM) for 24 h, and PLIN2 expression was determined by immunofluorescence staining, PLIN2 (green), DAPI (blue). Representative fluorescent images of the cells are shown, and quantitative data are shown in the right panels (Scale bars: 20 µm). **(D)** Proteins were extracted from the livers of mice administered with vehicle or diclofenac (100 mg/kg), and the levels of PLIN2 and GAPDH were analyzed by western blotting (n = 6). The bottom panels show the quantification of the PLIN2 and GAPDH levels. **(E)** mRNA was extracted from MPH treated with the indicated concentrations of diclofenac for 24 h, and mRNA levels of GAPDH and PLIN2 were analyzed by qRT-PCR. **(F)** MPH were treated with diclofenac (300 µM) and/or oleate (100 µM), and the levels of PLIN2 were analyzed by western blotting. The densitometric quantification of PLIN2 is shown in the right panels. Data are presented as mean ± SD of at least 3 independent experiments, as analyzed by one-way ANOVA followed by Tukey's test. *p < 0.05, **p < 0.01, and ***p < 0.001, relative to the control group. ##p < 0.01, relative to the oleate-treated group. n.s.; non-significant relative to the control group.

To investigate whether the lipid-accumulating effects of NSAIDs are associated with CMA activity, the expression of CMA substrate was monitored by Western blot analysis. We found a dose-dependent increase not only in PLIN2 but also in GAPDH following treatment with diclofenac in MPH, HepG2 and AML12 cells (Fig. 2A, Fig. S1D-E). Similar results were obtained from HepG2 cells treated with other NSAIDs (Fig. 2B; Fig. S1F). Fluorescence microscopy analysis demonstrated that diclofenac increased the mean staining intensity of PLIN2 and enlarged the size of PLIN2-surrounded LDs (Fig. 2C). Higher expression levels of PLIN2 and GAPDH were observed in the liver lysate from the diclofenac-treated mice relative to the control (Fig. 2D). However, diclofenac treatment did not change the mRNA levels of PLIN2 and GAPDH (Fig. 2E, Fig S1G). Moreover, the increase of PLIN2 by diclofenac was also observed under the lipogenic stimulus condition induced by oleate (Fig. 2F, Fig. S1H). Together, these findings indicate that NSAID treatment increases abundance of CMA substrate proteins in hepatocytes, which fact suggests the possibility of CMA inhibition by NSAIDs.

### NSAIDs inhibit CMA by reducing levels of LAMP2A in lysosomes

**Figure 3:**
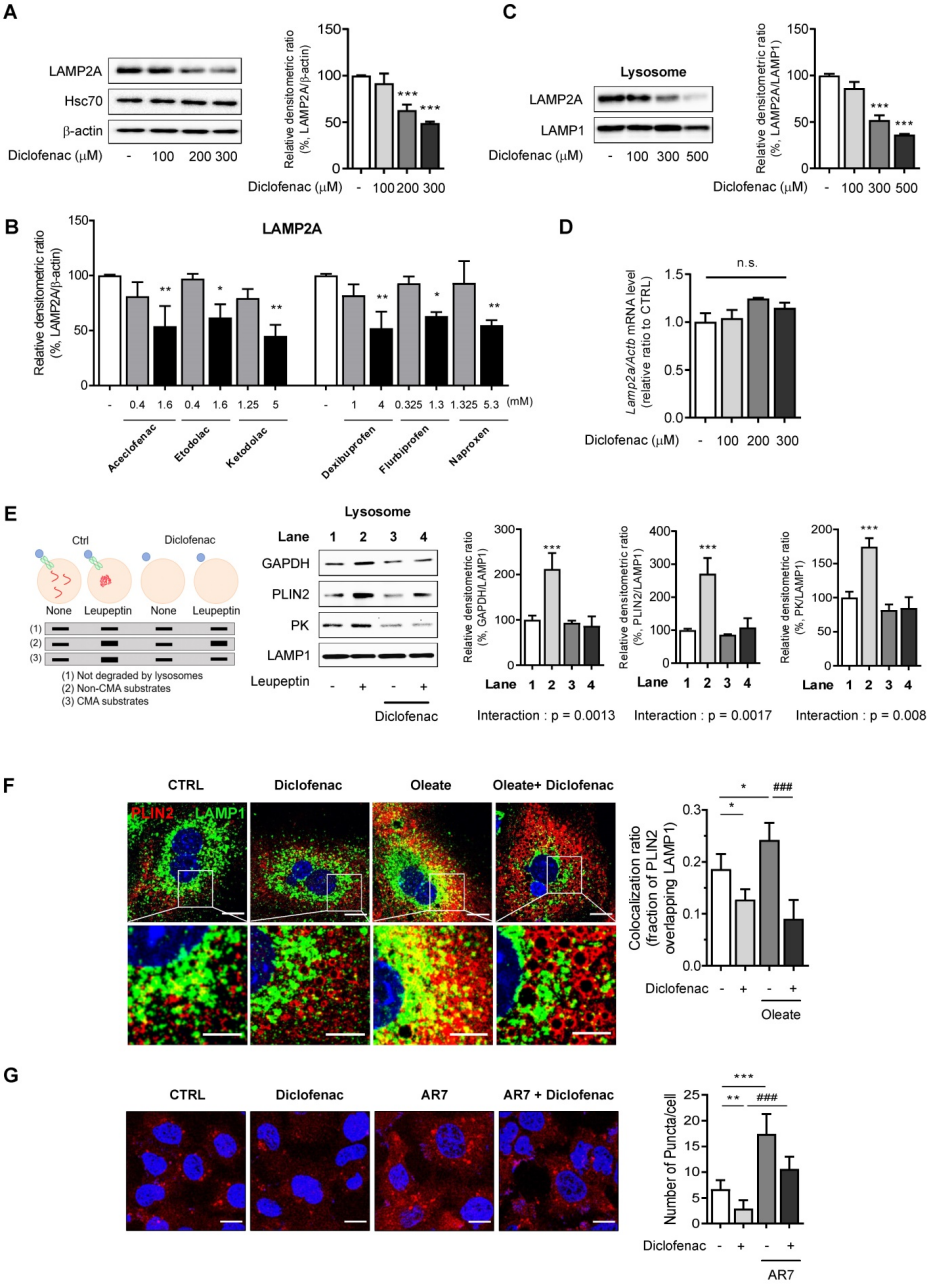
** NSAIDs inhibit CMA by reducing levels of LAMP2A in lysosome. (A)** Western blot analysis of LAMP2A in MPH treated with the indicated concentration of diclofenac for 24 h. The right panel shows the quantification of the LAMP2A level. **(B)** HepG2 cells were treated with the indicated concentration of NSAIDs for 24 h, and the levels of LAMP2A were analyzed by western blotting. **(C)** LAMP2A and LAMP1 were measured in lysosomal fractions isolated from diclofenac-treated HepG2 cells. The right panel shows the quantification of the LAMP2A level. **(D)** mRNA was extracted from MPH treated with the indicated concentration of diclofenac for 24 h, and mRNA levels of LAMP2A were analyzed by qRT-PCR. **(E)** Hypothetical changes in protein levels of lysosomes isolated from HepG2 cells treated with diclofenac (300 µM) and leupeptin (100 µM). The indicated CMA substrates (GAPDH, PLIN2, PK) were determined in the lysosomal fraction using western blotting. Two-way ANOVA was performed and the interaction effect is shown below the graphs **(F)** MPH were treated with diclofenac (300 µM) and oleate (100 µM) for 24 h, and PLIN2 and LAMP1 expression were determined by immunofluorescence staining. PLIN2 (red), LAMP1 (green), DAPI (blue) (Scale bars; 20 µm). Representative fluorescent images of the cells and the zoomed images magnified from the small boxed areas in each image are shown (Scale bars; 10 µm). Percentages of co-localization of PLIN2 with LAMP1 are shown in right panels. **(G)** HepG2 cells were treated with diclofenac (300 µM) and AR7 (10 µM) for 24 h followed by transfection with pSIN-PAmCherry-KFERQ-NE plasmid. Representative fluorescent images and quantification of PAmCherry-KFERQ puncta are shown (Red puncta; PAmCherry, Blue; DAPI). Data are presented as mean ± SD of at least 3 independent experiments, as analyzed by one-way ANOVA followed by Tukey's test. Figure 3E was analyzed by two-way ANOVA followed by Tukey's test. *p < 0.05, **p < 0.01, and ***p < 0.001, relative to the control group. ###p < 0.001, relative to the indicated group. n.s.; non-significant relative to the control group.

In order to test the hypothesis that NSAIDs inhibit CMA, we determined the level of LAMP2A, the main effector of CMA. We found that NSAIDs decreased the expression of LAMP2A in MPH, HepG2 and AML12 cells in a concentration-dependent manner without any change in the level of HSC70 (Fig. 3A, Fig. S2A-B). Similar results were obtained in HepG2 cells treated with other NSAIDs (Fig. 3B, Fig. S2C). Diclofenac treatment markedly decrease LAMP2A protein levels in lysosomal fractions. (Fig. 3C, Fig. S2D). The protein expression level of LAMP2A diminished over time without changes in the mRNA level, which suggests the possibility that diclofenac may abrogate the stability of LAMP2A (Fig. 3D, Fig. S2E-F). Next, to determine whether the inhibition of CMA by diclofenac suppressed the degradation of CMA substrates, we performed a comparative analysis of the lysosomal levels of the CMA substrates in cells in the absence or presence of leupeptin to block lysosomal proteolysis 20. As expected, the CMA substrates, GAPDH, PLIN2 and pyruvate kinase (PK) 21 were accumulated in the lysosome of leupeptin-treated cells and did not increase in the lysosome by the cotreatment with diclofenac and leupeptin (Fig. 3E). These data indicate that blockade of LAMP2A-mediated lysosomal uptake by diclofenac hinders the degradation of PLIN2.

To address whether the inhibition of CMA by diclofenac resulted in decreased CMA substrate translocation, we examined the colocalization of PLIN2 with LAMP1, a marker of lysosomes and late endosomes, in the absence and presence of diclofenac and/or oleate. The colocalization fraction of PLIN2 overlapping the lysosomal marker was decreased after exposure to diclofenac under both normal and lipogenic conditions (Fig. 3F). Finally, we evaluated the effects of diclofenac on CMA activity using PAmCherry-KFERQ, a photoactive CMA reporter that tracks their lysosomal uptake 22. The number of fluorescent-puncta significantly decreased after exposure to diclofenac when compared with control group. The CMA inhibitory effect of diclofenac was confirmed also under the condition of CMA activation by AR7, a RARα antagonist known to specifically activate CMA without affecting macroautophagy 23 (Fig. 3G).

To rule out the possibility that these effects of NSAID are linked to the inhibition of cyclooxygenase (COX), we used ibuprofen isomers to determine correlation between COX inhibition and CMA activity. We compared the effects of (S)- and (R)-ibuprofen, which are active and inactive on COX enzyme, respectively. We found that both stereoisomers and racemic mixture of ibuprofen accumulated intracellular lipid and inhibited CMA to a similar degree (Fig. S3A-B). Moreover, no significant change of CMA marker proteins was observed when *COX1* gene was knockdown in MPH (Fig. S3C). These results suggest that COX inhibitory activity is not responsible for the CMA impairment by NSAID. Collectively, these data demonstrate that the NSAIDs tested in this study inhibited CMA by lowering the level of LAMP2A in the lysosome of hepatocytes, leading to decreased lysosomal translocation of PLIN2.

### CMA reactivation reduces diclofenac-induced lipid accumulation in hepatocytes

**Figure 4:**
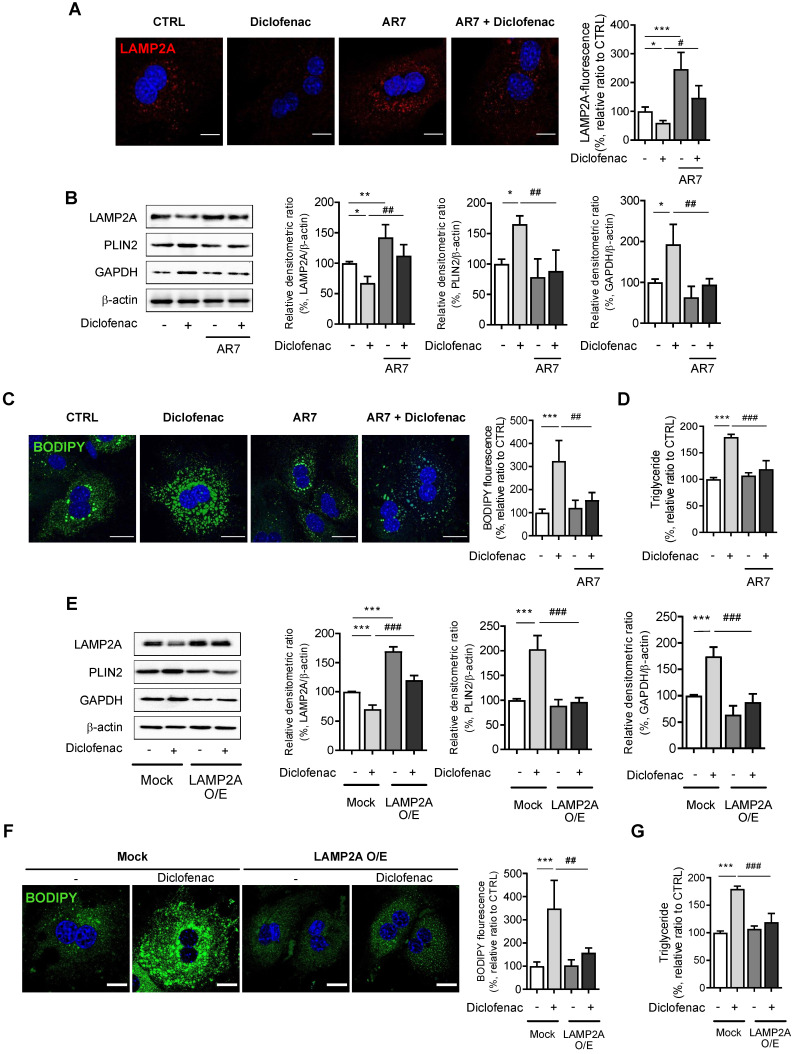
** CMA activation reduces diclofenac-induced lipid accumulation in hepatocytes. (A)** MPH were incubated with diclofenac (300 µM) or AR7 (10 µM) for 24 h. Confocal microscopy images was analyzed to detect LAMP2A. Representative fluorescent images and quantitative data of the cells are shown (Scale bars: 20 µm). **(B)** The protein levels of LAMP2A, PLIN2, and GAPDH were measured in MPH by western blot analysis. Representative western blot images and the densitometric quantification of proteins are shown. **(C)** MPH were incubated with diclofenac (300 µM) and AR7 (10 µM) for 24 h. Intracellular lipid droplets (LDs) were determined by BODIPY 493/503 fluorescence (green). Nuclei were stained with DAPI (blue) (Scale bars: 20 µm). **(D)** TG concentration in MPH was quantified using an enzymatic kit. **(E)** MPH were transfected with pCMV-LAMP2A plasmid and were incubated with diclofenac for 24 h. Western blot analysis with antibodies against LAMP2A, PLIN2 and GAPDH and the densitometric quantification of the PLIN2 and GAPDH level are shown. **(F)** Intracellular LDs were determined by BODIPY 493/503 fluorescence (green). Nuclei were stained with DAPI (blue) (Scale bars: 20 µm). **(G)** TG concentration in MPH was quantified using the enzymatic kit. Data are presented as mean ± SD of at least 3 independent experiments, as analyzed by one-way ANOVA followed by Tukey's test. *p < 0.05, **p < 0.01, and ***p < 0.001, relative to the control group. #p < 0.05, ##p < 0.01, ###p < 0.001, relative to the indicated group.

Next, we investigated whether reactivation of CMA reverses diclofenac-induced lipid accumulation in hepatocytes. For this, we treated the cells with AR7 or transfected with a LAMP2A overexpression vector. When MPH, HepG2 and AML12 cells were treated with AR7 alone or in combination with diclofenac, the expression of LAMP2A was significantly increased compared with cells treated with vehicle or diclofenac alone, respectively (Fig. 4A-B, Fig. S4A-B). AR7 reversed the diclofenac-induced failure in the degradation of CMA substrates such as PLIN2 and GAPDH (Fig. 4B, Fig. S4A-B). Furthermore, the determination of cytosolic neutral lipid using BODIPY493/503 fluorescence dye or by biochemical analysis revealed that AR7 treatment markedly decreased the lipid accumulation induced by diclofenac (Fig. 4C-D). Similar results were obtained in cells transfected with a LAMP2A overexpression vector. Overexpression of LAMP2A restored the GAPDH and PLIN2 degradation and reversed the lipid accumulation induced by diclofenac treatment (Fig. 4E-G, Fig. S4C). Reportedly, LDs are also susceptible to degradation by macroautophagy 2. Since we previously reported that NSAIDs inhibit macroautophagy as well, we investigated whether inhibition of macroautophagy also contribute to diclofenac-induced lipid accumulation 13. To investigate whether restoration of macroautophagic activity has any effects on diclofenac-induced lipid accumulation, we used clioquinol and rapamycin, both of which have been proven to activate autophagic flux in diclofenac-treated cells 13. Unlike AR7, however, treatment of clioquinol did not reverse the expression level of the LAMP2A or CMA substrates, and neither did it reverse lipid accumulation by diclofenac (Fig. S5A-B). In the experiment using rapamycin, the results were not much different (Fig. S5C-D). These data indicate that diclofenac-induced lipid accumulation is independent of non-CMA autophagy. Together, these results reveal that the incurring of defect in LAMP2A-mediated CMA activity is a major factor underlying the lipid accumulation by NSAIDs.

### Diclofenac decreases LAMP2A level via SNX10-mediated CTSA maturation

**Figure 5:**
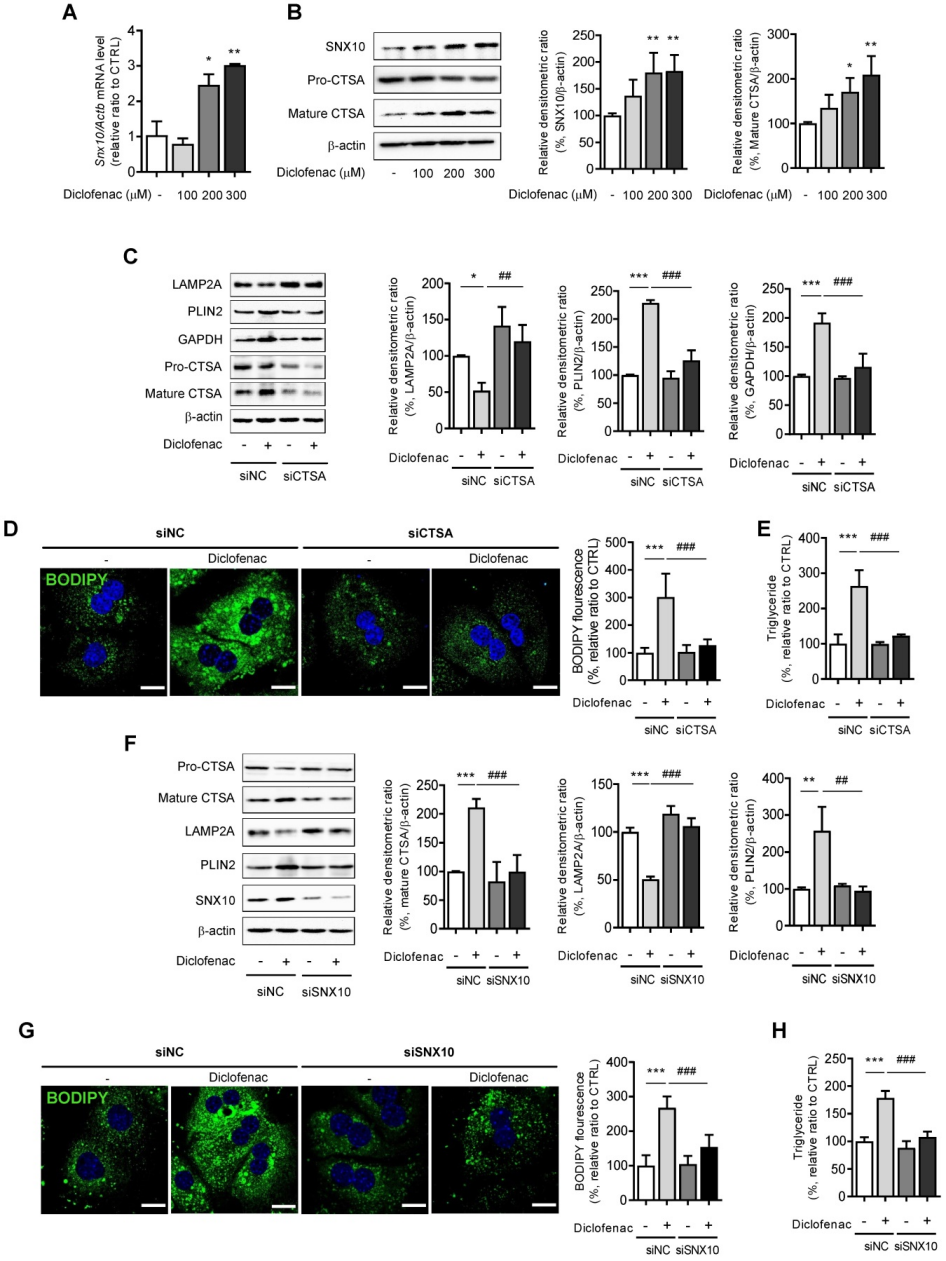
** Diclofenac decreases level of LAMP2A via SNX10-mediated activation of CTSA maturation. (A)** MPH were incubated with diclofenac (300 µM) for 24 h, and mRNA was isolated and subjected to qRT-PCR analysis to measure sorting nexin 10 (SNX10) mRNA expression. **(B)** The level of SNX10, pro-CTSA, and mature CTSA was measured by western blot analysis in MPH treated with diclofenac. Representative western blot images and the densitometric quantification of proteins are shown. **(C, D, E)** MPH were transfected with control or CTSA siRNA. (C) The protein levels of LAMP2A, PLIN2, GAPDH, pro-CTSA and mature CTSA were measured by western blot analysis. Representative western blot images and the densitometric quantification of proteins are shown. (D) Intracellular LDs were determined by BODIPY 493/503 fluorescence (green). Nuclei were stained with DAPI (blue) (Scale bars: 20 µm). (E) TG concentration was quantified using an enzymatic kit. **(F, G, H)** MPH were transfected with control or SNX10 siRNA. (F) The protein levels of LAMP2A, PLIN2, pro-CTSA, mature CTSA and SNX10 were measured by western blot analysis. Representative western blot images and the densitometric quantification of proteins are shown. (G) Intracellular LDs were determined by BODIPY 493/503 fluorescence (green). Nuclei were stained with DAPI (blue) (Scale bars: 20 µm). (H) TG concentration was quantified using the enzymatic kit. Data are presented as mean ± SD of at least 3 independent experiments, as analyzed by one-way ANOVA followed by Tukey's test. *p < 0.05, **p < 0.01, and ***p < 0.001, relative to the control group. ##p < 0.01, ###p < 0.001, relative to the diclofenac-treated group.

Next, we investigated the mechanism by which diclofenac decreases LAMP2A protein levels. We and others have already reported that diclofenac increases mitochondrial ROS, which is responsible for the suppression of autophagic flux 11 24. Another previous study demonstrated that diclofenac impairs autophagic flux via oxidative stress 13. To investigate whether ROS generated by diclofenac has any effect on CMA, we measured the parameters of CMA following co-treatment with ROS scavengers. Although N-acetylcysteine or Mito-tempo reversed the autophagy that is suppressed by diclofenac, the levels of the proteins associated with CMA were not altered in MPH (Fig. S5E-F). Based on the results that the transcription levels of LAMP2A were not changed by diclofenac treatment (Fig. 3D), we hypothesized that the protein stability of LAMP2A might be affected. Active CTSA interacts with LAMP2A on the lysosomal membrane and stimulates its degradation. And, according to You *et al.*, deficiency of SNX10 increases the stability of LAMP2A by inhibiting the trafficking and maturation of CTSA in the lysosome 8. As can be seen in Fig. 5A and B, diclofenac treatment increased the expression of SNX10 and the mature form of CTSA. To investigate the role of SNX10 in CMA activity and lipid accumulation by diclofenac, we used CTSA knockdown MPH. Decreased CMA activity and increased accumulation of CMA substrate as well as lipid induced by diclofenac treatment were completely abolished by knockdown of CTSA (Fig. 5C-E). The same was true in MPHs transfected with siRNA against SNX10. As expected, knockdown of SNX10 prevented diclofenac-induced maturation of CTSA, downregulation of LAMP2A and accumulation of PLIN2, which resulted in the inhibition of lipid accumulation (Fig. 5F-H). Taken together, these results indicate that inhibition of CMA and subsequent intracellular lipid accumulation by diclofenac is attributed to increased expression of SNX10, activation of CTSA, and, thus, degradation of LAMP2A.

### Endoplasmic reticulum (ER) stress is responsible for increase of SNX10 by NSAIDs

**Figure 6:**
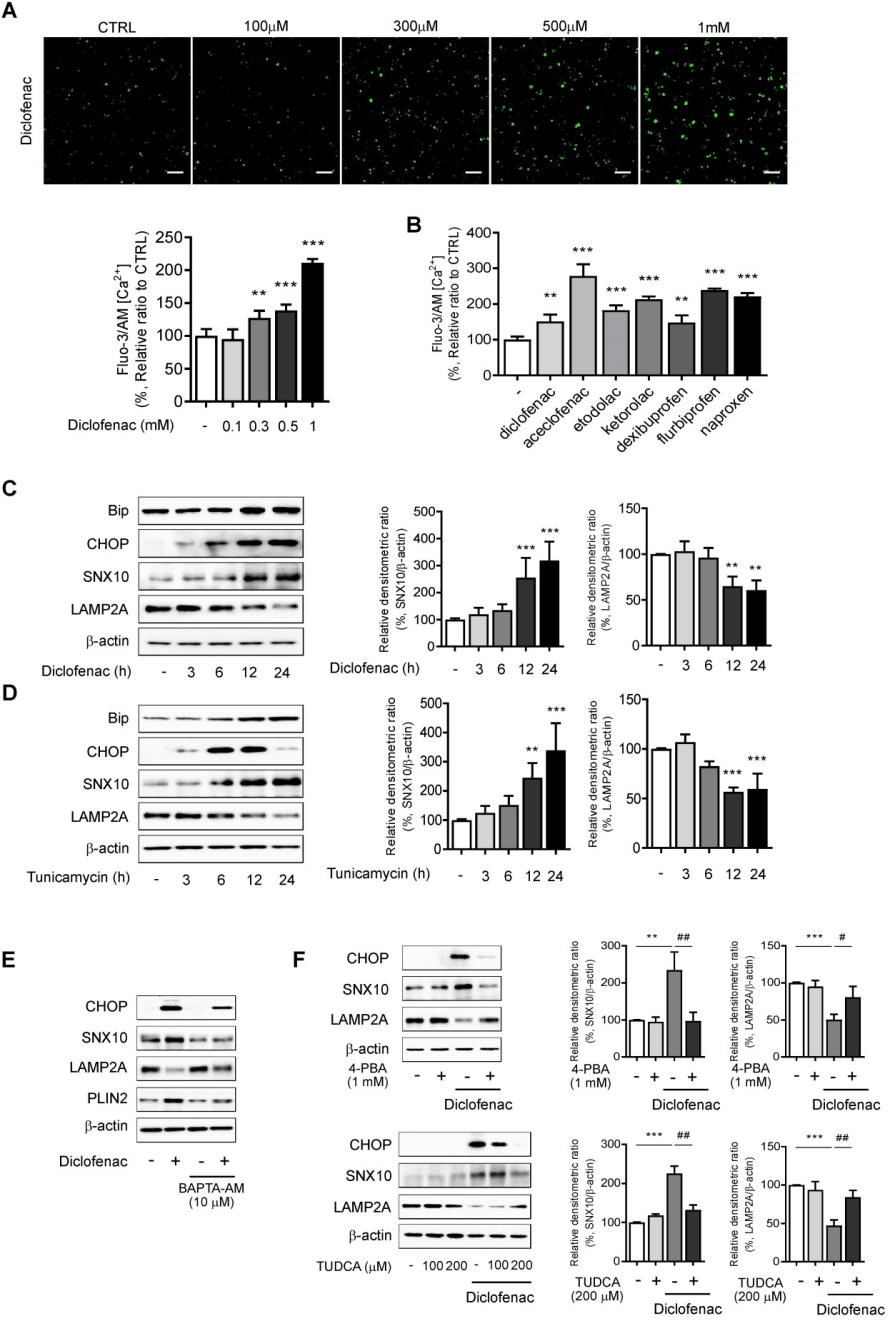
** ER stress is responsible for the increase of SNX10 by NSAIDs. (A)** HepG2 cells were incubated with the indicated concentration of diclofenac for 30 min, and intracellular Ca^2+^ level was analyzed by fluo-3 AM. The fluorescence intensity was calculated using an Incucyte cell imaging microplate reader (Scale bars: 100 µm). **(B)** HepG2 cells were treated with NSAIDs (Diclofenac; 0.5 mM, Aceclofenac; 1.6 mM, Etodolac; 1.6 mM, Ketodolac; 5 mM, Dexibuprofen; 4 mM, Flurbiprofen; 1.3mM, Naproxen; 5.3 mM) and intracellular Ca^2+^ level was analyzed by fluo-3 AM. The fluorescence intensity was calculated using an Incucyte cell imaging microplate reader. **(C, D)** HepG2 cells were treated with diclofenac (300 µM) or tunicamycin (3 µg/ml) at the indicated time point and subjected to western blot analysis with antibodies against Bip, CHOP, and SNX10 and LAMP2A and the densitometric quantification of the SNX10 and LAMP2A level are shown. **(E)** HepG2 cells were pretreated with BAPTA-AM (10 µM) for 1 h before diclofenac treatment (300 µM). The protein levels of CHOP, SNX10, LAMP2A and PLIN2 were measured by western blot analysis. **(F)** HepG2 cells were treated with diclofenac (300 µM) in the presence or absence of 4- phenylbutyrate (4-PBA, 1 mM) or taurodeoxycholic acid (TUDCA, 100, 200 µM; 2 h pretreatment) and subjected to western blot analysis with antibodies against CHOP, SNX10, and LAMP2A. The densitometric quantification of SNX10 and LAMP2A are shown in the right panels. Data are presented as mean ± SD of at least 3 independent experiments, as analyzed by one-way ANOVA followed by Tukey's test. **p < 0.01, and ***p < 0.001, relative to the control group. #p < 0.05, ##p < 0.01, relative to the diclofenac-treated group.

We explored the mechanisms by which diclofenac increases the expression of SNX10. We analyzed the transcription factor binding sites of SNX10 using QIAGEN (GeneCards.org) and the ChIPseq database on GTRD 25. C/EBP α/β and C/EBP-homologous protein (CHOP) were identified as possible candidates for SNX10 transcription factors (data not shown). Therefore, we hypothesized that endoplasmic reticulum stress responses might be associated with SNX10 expression. The ER stress response is involved in NSAID-induced cell death and toxicity 26, 27 where Ca^2+^-dependent CHOP induction plays an important role 28. To assess whether NSAIDs elevate intracellular Ca^2+^ level in liver cells, we treated NSAIDs on HepG2 cells loaded with fluo-3 AM, the calcium-binding fluorescent dye. The intracellular Ca^2+^ concentration was significantly increased by treatment of diclofenac in a dose-dependent manner (Fig. 6A). In fact, all of the CMA-inhibiting NSAIDs showed similar results (Fig. 6B). Diclofenac treatment upregulated the protein levels of the ER stress markers, the glucose-regulated protein-78 (GRP78/BiP) and CHOP together with those of SNX10 in HepG2 and MPH in a time-dependent manner (Fig. 6C, Fig. S6A). When the general ER stress responses were induced in the cells with tunicamycin, the expression of SNX10 was increased significantly concomitant with the lowered expression of LAMP2A (Fig. 6D, Fig. S6B). To determine whether increased intracellular Ca^2+^ is important for diclofenac-induced upregulation of SNX10, we used BAPTA-AM, a cell permeable intracellular Ca^2+^ chelator. As shown in Fig. 6E, BAPTA-AM alleviated diclofenac-induced ER stress response, SNX10 expression and CMA inhibition. We further confirmed the effect of diclofenac-induced ER stress on SNX10 and LAMP2A expression using ER stress inhibitors, 4-phenyl butyrate and taurodeoxycholic acid. Co-treatment with the inhibitor with diclofenac reversed the change of diclofenac-induced LAMP2A and SNX10 expression (Fig. 6F).

In contrast to previous findings, Li *et al.* reported that ER stress induces the CMA pathway in SN4741 cells (a nigral dopaminergic cell line) by recruiting mitogen-activated protein kinase 4 (MKK4) to phosphorylate LAMP2A for CMA activation 29. Therefore, the levels of SNX10 and LAMP2A were evaluated in MPH and SN4741 cells after treatment of diclofenac or tunicamycin. The expression of SNX10 was deficient in SN4741 cells. Diclofenac or tunicamycin downregulated the level of LAMP2A in MPH. However, the LAMP2A level was upregulated in SN4741 cells (Fig. S7A-B).

**Figure 7:**
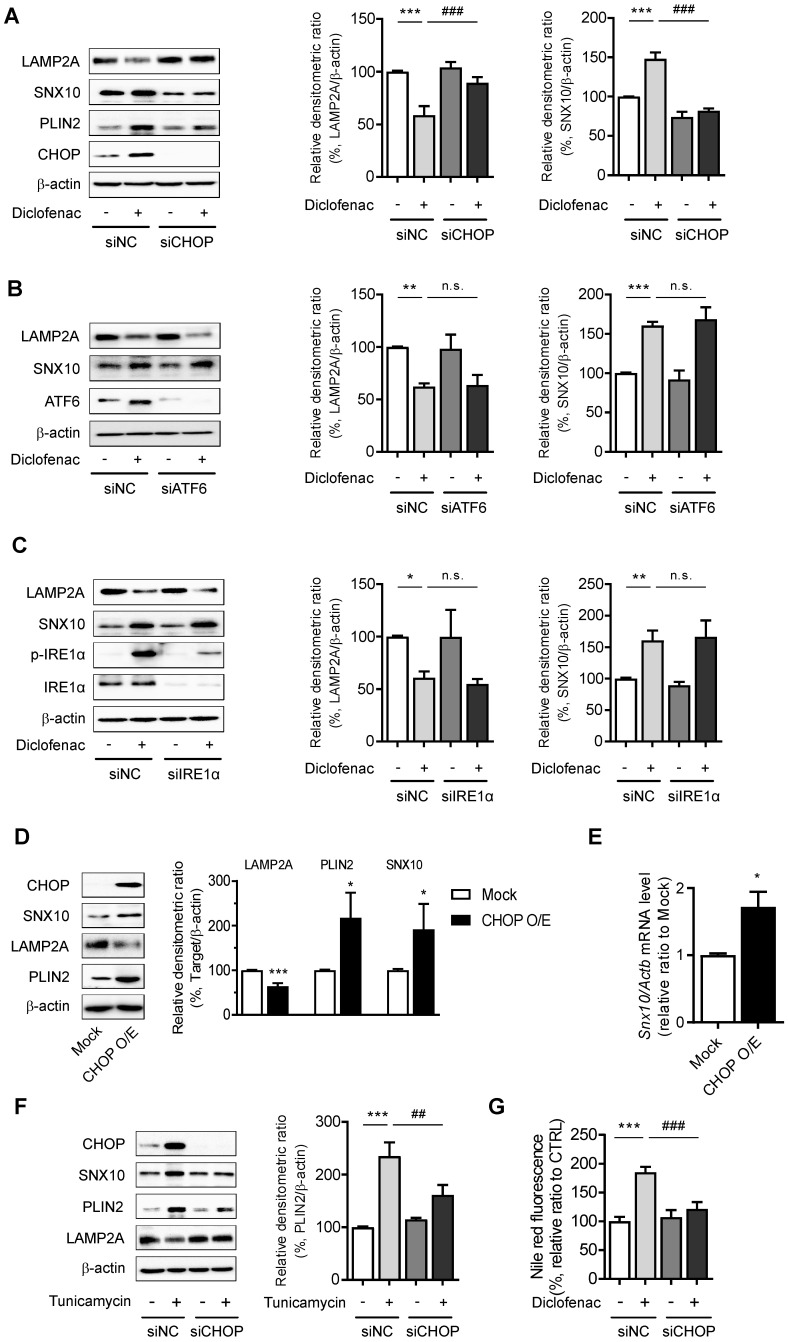
** CHOP expression mediates SNX10 upregulation and diclofenac-induced inhibition of CMA. (A, B, C)** Western blot for LAMP2A, PLIN2 and SNX10 after knockdown of each UPR pathway. HepG2 cells were treated with diclofenac (300 µM) for 24 h after transfection with control siRNA or each specific siRNA against CHOP (A), ATF6 (B), or IRE1α (C). The densitometric quantification of LAMP2A and SNX10 are shown in the right panels. **(D, E)** HepG2 cells were transfected with pCMV-CHOP plamsid. (D) The protein levels of CHOP, SNX10, LAMP2A, PLIN2 were measured by western blot analysis. (E) Relative mRNA levels of SNX10 were analyzed by qRT-PCR. **(F)** HepG2 cells were transfected with control or CHOP siRNA and incubated with tunicamycin (3 µg/ml) for 12 h. The protein levels of CHOP, SNX10, PLIN2 and LAMP2A were measured by western blot analysis. The densitometric quantification of PLIN2 is shown in the right panel. **(G)** HepG2 cells were transfected with control or CHOP siRNA and incubated with diclofenac (300 µM) for 24 h. Intracellular lipid concentrations were quantified using Nile-red dye; the excitation and emission wavelengths were 486 and 528 nm, respectively. Data are presented as mean ± SD of at least 3 independent experiments, as analyzed by one-way ANOVA followed by Tukey's test. *p < 0.05, **p < 0.01, and ***p < 0.001, relative to the control group. ##p < 0.01, relative to the tunicamycin-treated group. ###p < 0.001, relative to the diclofenac-treated group. n.s.; non-significant relative to the control group.

To further identify the pathway that mediates the ER stress-induced CMA inhibition, the main mediators of unfolded protein response (UPR) were inhibited by using siRNA of CHOP, activating transcription factor 6 (ATF6), or inositol-requiring transmembrane kinase/endoribonuclease 1α (IRE1α). Knockdown of CHOP reversed the change of diclofenac-induced SNX10, PLIN2 and LAMP2A expression, although the knockdown of ATF6 or IRE1α had no effect on SNX10 and LAMP2A levels (Fig. 7A-C). Indeed, overexpression of CHOP increased the expression of SNX10 and inhibited the activity of CMA levels (Fig. 7D-E). Knockdown of CHOP also masked the effect of tunicamycin on SNX10, PLIN2 and LAMP2A protein (Fig. 7F). Knockdown of CHOP rescued the number of PAmCherry-KFERQ reporter puncta per cell reduced by diclofenac or tunicamycin treatment (Fig. S8A-B). Knockdown of CHOP reversed the lipid accumulation by diclofenac completely, confirming that CHOP-dependent SNX10 induction is ultimately responsible for the lipid accumulation by diclofenac (Fig. 7G). These data indicate that CHOP-dependent ER stress response induces SNX10 induction resulting in the CMA inhibition and thus lipid accumulation.

### CMA activator alleviates diclofenac-induced hepatic steatosis *in vivo*

To establish the role of CMA-dysregulation in diclofenac-induced hepatic steatosis *in vivo*, 7-week-old C57BL/6 mice were intraperitoneally injected with diclofenac and/or AR7.

**Figure 8:**
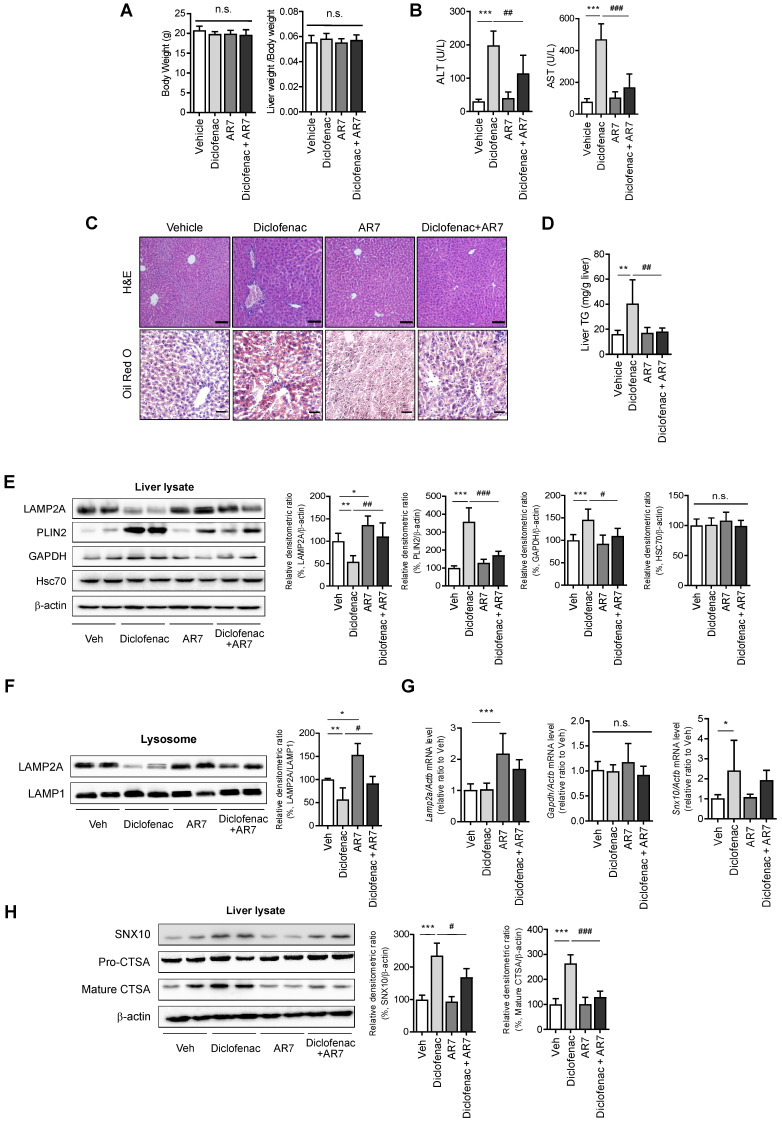
** CMA activator alleviates diclofenac-induced hepatic steatosis *in vivo*.** Male C57BL/6 mice were injected intraperitoneally with AR7 (10 mg/kg) 1 h before diclofenac (100 mg/kg) administration twice every 12h and sacrificed after the final injection. **(A)** The body weight and liver/body weight ratio are presented. **(B)** Serum ALT, AST measurements were performed using an automated Chemistry Analyzer (Tokyo Boeki Medical system, Prestige 24I) **(C)** Histological images of H&E (Scale bars; 100 µm, x20 magnification) and Oil Red O staining of mice liver (Scale bars; 50 µm, x40 magnification). **(D)** The level of hepatic TG (mg/g liver) is presented. **(E)** Protein extracts were prepared from mouse liver homogenates, and the protein levels of LAMP2A, PLIN2, GAPDH, and HSC70 were assessed by western blotting. The right panel shows the densitometric quantification of target proteins. **(F)** Western blots for LAMP2A protein in a lysosomal fraction of mice liver. **(G)** mRNA was prepared from mouse livers, and the mRNA levels of LAMP2A, GAPDH, SNX10 were assessed by qRT-PCR. **(H)** Protein extracts were prepared from the liver homogenates and subjected to western blot analysis with antibodies against SNX10, Pro-CTSA, cleaved CTSA. The densitometric quantification of proteins is shown. All of the data are mean ± SD of 6 mice per group in *in vivo* experiments, as analyzed by one-way ANOVA followed by Tukey's test. *p < 0.05, **p < 0.01, and ***p < 0.001, relative to the control group. #p < 0.05, ##p < 0.01, ###p < 0.001, relative to the indicated group. n.s.; non-significant relative to the control group.

Although there was no difference in body weight or liver-to-body weight ratio (Fig. 8A), the serum ALT and AST levels revealed significant damage in diclofenac-exposed livers. Co-administration of AR7 abrogated the increase of ALT and AST significantly (Fig. 8B). Hepatic lipid accumulation measured by Oil Red O staining and biochemical analysis of TG revealed significant alleviation of hepatic steatosis in the diclofenac- and AR7-injected mice (Fig. 8C-D). Of note, the expression levels of the CMA substrates and LAMP2A were reversed by the administration of AR7 with diclofenac. The levels of Hsc70 did not change in any of the groups (Fig. 8E). LAMP2A from the liver lysate, as well as from the purified lysosomal fraction, was downregulated by diclofenac injection (Fig. 8F). As expected, the mRNA levels of LAMP2A and GAPDH were not changed by the administration of diclofenac. On the other hand, the primary target of diclofenac, SNX10, was significantly increased at the transcriptional level by diclofenac administration, which was reversed by AR7 only slightly, not significantly (Fig. 8G). Expression of SNX10 was increased by diclofenac, followed by elevation of SNX10-mediated CSTA maturation. Administration of AR7 with diclofenac blocked the escalation of SNX10, as well as a mature form of CSTA (Fig. 8H).

Taken together, these data indicate that diclofenac induces SNX10 expression and maturation of CTSA, which lead to LAMP2A degradation and suppression of CMA activity. Impaired CMA failed to degrade CMA substrates such as PLIN2, which ultimately caused diclofenac-induced hepatic steatosis and toxicity.

## Discussion

Lipids are not solely a component of cellular energy storage or membrane structure; they also function as signaling molecules in cellular response pathways. Disruption of homeostasis in lipid metabolism, therefore, leads to diverse organ diseases including neuronal, metabolic, and immunological dysfunctions. Lipids are degraded by lipolysis and autophagy during times of nutrient deprivation. While LDs in adipocytes are hydrolyzed predominantly by lipases, their expression in non-adipose tissue is relatively low. Therefore, the lysosomal-autophagic pathway plays a critical role in the early steps of lipid degradation in hepatocytes 30. Selective degradation of PLIN2 by CMA and subsequent activation of lipolysis or lipophagy are critical cellular pathways of LD breakdown. Earlier studies have shown that pirprofen and ibuprofen inhibit microsomal β-oxidation and thereby induce haptic steatosis 14, 15. However, it has not been reported whether toxicity also occurs in other NSAIDs or what the underlying molecular mechanism is or might be. We reported recently that NSAID-induced lysosomal dysfunction downregulates cellular autophagic flux, which induces an impairment of mitochondrial quality control and thus hepatotoxicity 13. Investigating the possible association of NSAID-induced hepatic abnormal lipid metabolism with autophagy, we found that all of the NSAIDs tested in that study induced significant neutral lipid accumulation in hepatocytes, and that diclofenac was the most potent. We determined that diclofenac and other structurally related NSAIDs inhibit CMA by enhanced degradation of LAMP2A in the lysosome, which prevents efficient degradation of PLIN2, leading to cellular lipid accumulation and hepatic steatosis. Diclofenac increased the expression of CHOP via the ER stress pathway. The CHOP-mediated upregulation of SNX10 accelerated the degradation of LAMP2A via the maturation of CTSA. In this paper, we report for the first time that NSAIDs induce lipid accumulation in the liver through CMA inhibition. The results presented herein highlight the role of CMA in drug-induced liver injury and offer new insight into the molecular mechanisms of NSAID-induced hepatotoxicity, including steatosis.

CMA is a selective form of autophagy targeting only proteins with a motif recognized by the HSC70 chaperone. The substrate-chaperone complex binds the lysosomal receptor LAMP2A and the substrate proteins are translocated into the lysosomal lumen where they undergo rapid degradation 31. CMA activity is determined by the lysosomal level of LAMP2A 32. In the liver, the basal level of lysosomal LAMP2A and starvation-induced CMA activity is relatively high 33. Therefore, basal and induced CMA activity in the liver is important for the maintenance of lipid homeostasis. Conditional knockout of LAMP2A in mouse liver reduces the degradation of lipid regulatory enzymes, thereby leading to profound hepatic steatosis 6. Likewise, peritoneal macrophages isolated from macrophage-specific LAMP2A-deficient mice exhibit significant intracellular lipid accumulation 34. Conversely, inhibition of CMA has been observed in various metabolic liver diseases. Jose *et al.* reported that high-lipid-content diets blocked CMA activity by facilitating lysosomal LAMP2A degradation 35. Moreover, liver tissues from patients with non-alcoholic fatty liver disease (NAFLD) exhibited a negative relationship between LAMP2A expression and NAFLD steatosis grade 36. Sharma *et al.* confirmed that in primary human hepatocytes' response to fatty acid treatment, the LAMP2A level was reduced 37. These findings indicate that reduced CMA activity induces dysregulation of lipid metabolism and vice versa.

In contrast to the many reports on compromised CMA activity under pathophysiological conditions, only a few have been published on changes in CMA activity following treatment with toxic chemicals. CMA marker proteins including LAMP2A and HSC70 were significantly downregulated in hepatocytes isolated from rats with D-galactosamine/lipopolysaccharide-induced acute liver failure; CMA was restored through PI3K inhibition and normal liver function was recovered 38. Conflicting results have been published on the effect of ethanol feeding on CMA. Cai *et al.* reported suppression of CMA activity through reduction of HSC70 and LAMP2A in the liver of mice fed the Lieber-DeCarli ethanol diet for 4 weeks 39; You *et al.* published the opposite results 9. The factors behind this discrepancy are unknown, as there were no differences in experimental conditions regarding ethanol concentration, duration of feeding, or animal species used. Even so, You *et al.*'s further activation of CMA by increasing the stability of LAMP2A through liver-specific SNX10 deletion alleviated alcohol-induced liver injury and steatosis 9. In the present study, we determined, definitively, that diclofenac and other structurally related NSAIDs inhibit CMA by enhanced degradation of LAMP2A in the lysosome. We also demonstrated, for the first time, that suppression of CMA activity contributes to hepatic lipid accumulation induced by NSAID treatment. Our overall results can be considered to reveal the essential mechanism by which NSAIDs induce hepatic lipid accumulation. This is the first reporting of an essential mechanism since fatty acid beta-oxidation inhibition was revealed to be a mechanism of NSAID-induced hepatic steatosis 30 years ago 15.

According to the recent report by Endicott *et al.*, CMA regulates lipid accumulation by regulating the abundance of the enzymes responsible for lipid as well as nucleocytosolic acetyl-CoA synthesis 40. On the other hand, the decrease of LAMP2A by diclofenac treatment did not change the expression of lipogenic genes in our study (Fig. S1D). Moreover, to date, there has been no report that NSAIDs affect acetyl-CoA production and *de novo* lipogenesis. We consider the difference originates from the duration and the intensity of CMA inhibition. Further studies merit to be carried out to elucidate the issue clearly.

The CMA process is tightly regulated for maintenance of cellular homeostasis. As LAMP2A is the main effector of CMA, its abundance in the lysosomal membrane is used as an indirect indicator of CMA status. Some transcription factors, including nuclear factor of activated T cells or nuclear factor erythroid 2-related factor 2, have been described as modulators of LAMP2A expression 7, 41. LAMP2A trafficking into the lysosomal membrane or LAMP2A stability therein is the key post-transcriptional modulator of lysosomal LAMP2A level 8, 9, 42. In addition, the dynamics of the assembly and disassembly of the LAMP2A translocation complex regulated by the interaction between Akt and mTORC2/PHLPP1 are another mechanism of LAMP2A's functional regulation 43. Different CMA stimuli modify the LAMP2A level through still-other mechanisms. Increased LAMP2A under basal conditions is mainly due to *de novo* synthesis, but reduced breakdown of LAMP2A in the lysosomal membrane is the primary mechanism of increased LAMP2A levels in starvation. Because we could not detect differences in LAMP2A mRNA level upon treatment of diclofenac, we explored the possibility that NSAIDs manipulate the protein stability of LAMP2A. In a study by Cuervo *et al.*, a serine protease CTSA was identified as an enzyme responsible for LAMP2A degradation in lysosome and release of truncated LAMP2A into the matrix 8. Our study found that CTSA maturation by diclofenac mediates LAMP2A degradation and thus CMA inhibition *in vitro* and *in vivo*. Knockdown of CTSA restored LAMP2A expression and attenuated diclofenac-induced hepatic lipid accumulation. These data support the hypothesis that the NSAID-induced inhibitory effects on CMA result from CTSA-mediated degradation of LAMP2A in the liver.

The upstream target of diclofenac-induced CTSA maturation was found to be SNX10 transcription. SNX10 is one of the simplest-in-structure isoforms of the sorting nexin family, a family of evolutionarily conserved proteins involved in vesicular trafficking between cellular compartments 44. A recent study by You *et al.* demonstrated the role of SNX10 in alcohol-induced liver steatosis. They found that SNX10 deficiency alleviated alcoholic liver injury by activating CMA through upregulated transcription and stability of LAMP2A 9. This is in concordance with our data showing that increased expression of SNX10 by diclofenac ultimately led to hepatic steatosis. SNX10 expression is expected to be closely correlated with various fatty liver diseases. Microarray data has shown that the level of SNX10 is significantly elevated in the mice fed a methionine- and choline-deficient plus high-fat (MCD+HF) diet. The NCBI Gene Expression Omnibus (GEO) database has revealed that SNX10 is highly expressed in the human liver of NAFLD patients (accession numbers GDS4884, GSE48452) (Fig. S9) 45, 46. Moreover, it has been reported that the level of SNX10 is upregulated in human atherosclerotic lesions 47. A follow-up study is needed on whether increased SNX10 expression in various diseases is implicated in disease developing or exacerbation via CMA.

The lack of reports on the transcriptional regulation of SNX10 led us to analyze the ChIPseq database and to identify that CHOP, a transcription factor involved in the ER stress response, is a plausible regulator of SNX10 48. ER is responsible for the folding of newly synthesized proteins and the maintaining of intracellular Ca^2+^ homeostasis 49. In fact, previous studies have shown that ER stress is involved in NSAID-induced toxicity 26, 50, where increased intracellular Ca^2+^ level is associated with the upregulation of CHOP 28. In line with these results, our data confirmed that all of the NSAIDs tested in our study significantly elevated intracellular Ca^2+^ level and that diclofenac increased the expression of Bip and CHOP in a Ca^2+^-dependent manner. CHOP was essential for SNX10 expression, thereby inhibiting LAMP2A expression and CMA activity. Our results contradict the findings of Li *et al.* that ER stressors lead to the activation of CMA via p38 MAPK-mediated phosphorylation and stabilization of LAMP2A in the dopaminergic cell line SN4741 29. Indeed, the expression of SNX10 is deficient, and the level of LAMP2A is not downregulated by diclofenac and tunicamycin in SN4741 cells. These results suggest that SNX10 plays a role in the inhibition of CMA in the liver following ER stress induction, which in turn depends on the cell type and the cellular environment. Further studies are needed to clarify the exact role of ER stress in LAMP2A stability and CMA activity.

There exist compensatory mechanisms between CMA and macroautophagy. Deletion of Atg5, an autophagy-related protein required for autophagosome formation, leads to CMA's up-regulation, even under basal conditions 51. Similarly, chronic blockage of CMA by RNA interference against LAMP2A results in constitutive activation of macroautophagy 52. Thus, although CMA-deficient cells are more susceptible to various stresses, prevention of the compensatory activation of macroautophagy increases sensitivity, which indicates that up-regulation of macroautophagy in these cells contributes to maintenance of cellular homeostasis and viability 53. According to our present and previous studies, NSAIDs inhibit both macroautophagy and CMA in hepatocytes. The impairment of macroautophagy by NSAIDs, caused by intracellular ROS and lysosomal dysfunction, prevents efficient degradation of damaged mitochondria and, thus, minimization of additional ROS production 13. Concomitant defects of CMA by NSAIDs render cells more susceptible to oxidative stress 54. CMA inhibition by NSAIDs induces intracellular lipid accumulation by inhibiting degradation of the CMA substrate protein, PLIN2. This dual compromise of the two essential autophagy mechanisms may underlie the basis of cellular toxicity following exposure to NSAIDs. The current study demonstrated the role of CMA in progression of NSAID-induced hepatic steatosis. Our data indicate that upregulation of SNX10 activates accelerated CTSA maturation and impaired CMA-mediated degradation of lipid-associated protein, PLIN2. Together with the ubiquitin/proteasome system, these autophagy pathways are primarily responsible for cellular homeostasis. Therefore, compromise of the two autophagy pathways can make cells particularly vulnerable to stressors, as they cannot cope with stressor-related damage.

In summary, we demonstrated the role of CMA in progression of NSAID-induced hepatic steatosis. We found that upregulation of SNX10 activates accelerated CTSA maturation and impaired CMA-mediated degradation of lipid-associated protein, PLIN2. These results represent a novel insight connecting NSAID overdose to hepatic steatosis.

## Supplementary Material

Supplementary figures and table.

## Abbreviations

ATF6: Activating transcription factor 6
Bip (GRP78): binding immunoglobulin protein (the glucose-regulated protein-78)
BODIPY 493/503: 4,4-Difluoro-1,3,5,7,8-pentamethyl-4-bora-3a,4a-diaza-s-indacene
CHOP: C/EBP homologous protein
COX: cyclooxygenase
CMA: chaperone-mediated autophagy
CTSA: Cathepsin A
ER: endoplasmic reticulum
GAPDH: glyceraldehyde 3-phosphate dehydrogenase
HSC70: heat shock cognate 71 kDa protein
IRE1α: inositol-requiring transmembrane kinase/endoribonuclease 1α
LAMP: lysosomal associated membrane protein
LD: lipid droplet
MPH: mouse primary hepatocytes
NAFLD: non-alcoholic fatty liver disease
NSAID: non-steroidal anti-inflammatory drug
PLIN: perilipin
ROS: reactive oxygen species
SNX10: sorting nexin 10
TG: triglyceride
UPR: unfolded protein response
