# Supplementary material for: SNX10-mediated degradation of LAMP2A by NSAIDs inhibits chaperone-mediated autophagy and induces hepatic lipid accumulation
Source: Theranostics. 2022 Feb 21;12(5):2351–69. doi: 10.7150/thno.70692 (PMC8899581; doi:10.7150/thno.70692)
Supplement: Supplementary file 1 — Supplementary figures and table. [file thnov12p2351s1.pdf]

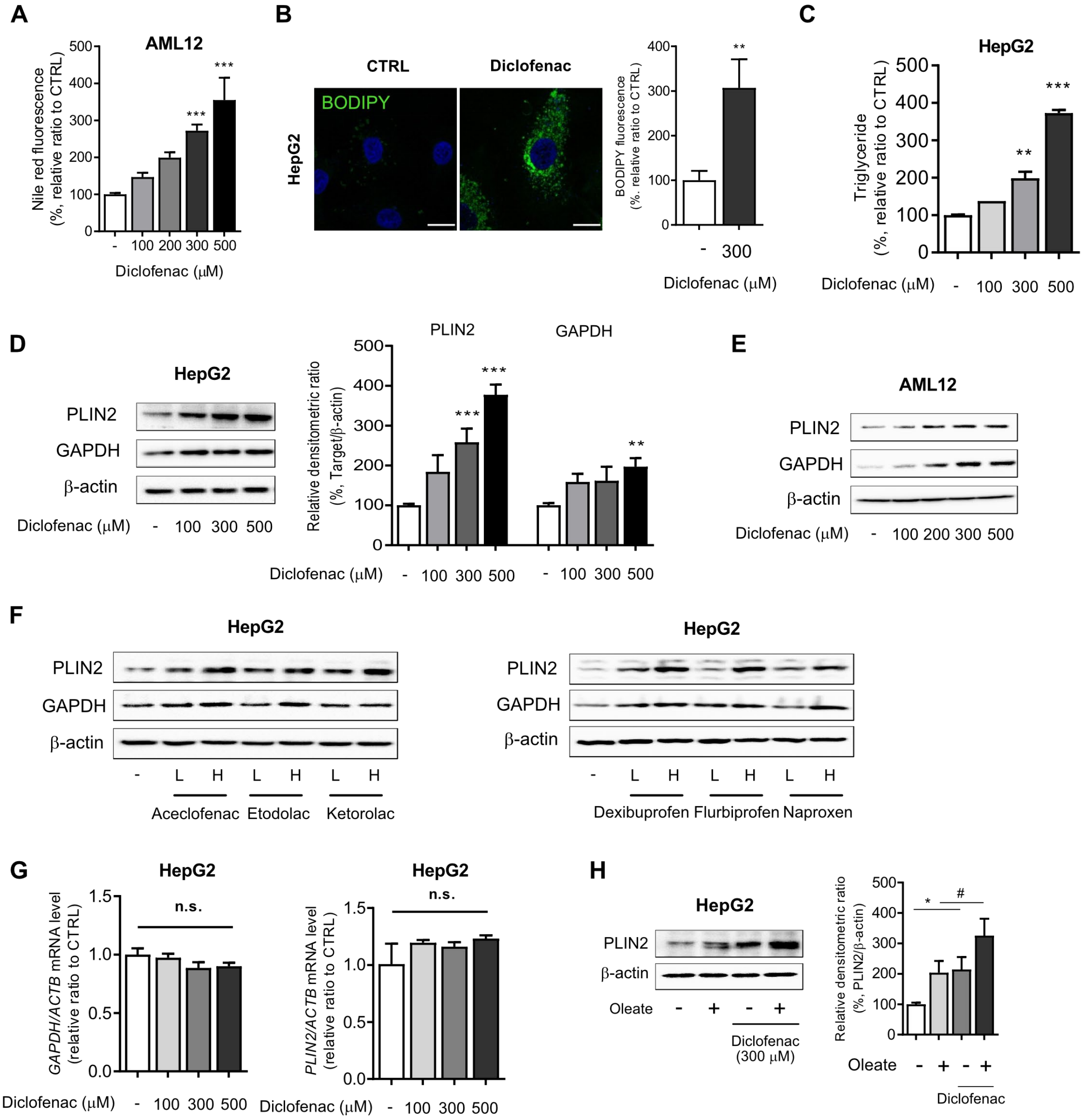
A
AML12
Nile red fluorescence (%, relative ratio to CTRL)
***
***
- 100 200 300 500
Diclofenac (μM)
B
CTRL
Diclofenac
BODIPY
HepG2
BODIPY fluorescence (%, relative ratio to CTRL)
**
- 300
Diclofenac (μM)
C
HepG2
Triglyceride (%, relative ratio to CTRL)
**
***
Diclofenac (μM) - 100 300 500
D
HepG2
PLIN2
GAPDH
β-actin
Diclofenac (μM) - 100 300 500
PLIN2
GAPDH
Relative densitometric ratio (%, Target/β-actin)
Diclofenac (μM) - 100 300 500 - 100 300 500
E
AML12
PLIN2
GAPDH
β-actin
Diclofenac (μM) - 100 200 300 500
F
HepG2
PLIN2
GAPDH
β-actin
- L H L H L H
Aceclofenac Etodolac Ketorolac
HepG2
PLIN2
GAPDH
β-actin
- L H L H L H
Dexibuprofen Flurbiprofen Naproxen
G
HepG2
n.s.
GAPDH/ACTB mRNA level (relative ratio to CTRL)
Diclofenac (μM) - 100 300 500
HepG2
n.s.
PLIN2/ACTB mRNA level (relative ratio to CTRL)
Diclofenac (μM) - 100 300 500
H
HepG2
PLIN2
β-actin
Oleate - + - +
Diclofenac (300 μM)
Relative densitometric ratio (%, PLIN2/β-actin)
*
#
Oleate - + - +
Diclofenac

Supplementary Figure 1. Diclofenac induces lipid accumulation and increase abundance of CMA substrate in murine normal hepatocyte (AML12) and in human hepatoma cells (HepG2).

(A) AML12 cells were treated with diclofenac at the indicated concentrations for 24 h and intracellular lipid concentrations were quantified using Nile-red dye; excitation and emission wavelengths of 486 and 528 nm, respectively. (B) HepG2 cells were treated with diclofenac (300 μM) and stained with BODIPY 493/503 (green). Nuclei were stained with DAPI (blue). Representative fluorescent images and quantitative data of the cells are shown (Scale bars: 20 μm). (C) HepG2 cells were treated with diclofenac at the indicated concentration for 24 h, and intracellular triglyceride (TG) concentrations were quantified using an enzymatic kit. (D) Western blot analysis of PLIN2 and GAPDH in HepG2 cells, treated with the indicated concentrations of diclofenac for 24 h. The right panels show the densitometric quantification of the PLIN2 and GAPDH levels. (E) Western blot analysis of PLIN2 and GAPDH in AML12 cells treated with the indicated concentrations of diclofenac for 24 h. (F) Representative western blot images in Figure 2B, C. HepG2 cells were incubated with the indicated concentration of several NSAIDs for 24 h. The protein levels of PLIN2, GAPDH were measured by western blot analysis. Representative western blot image are shown (L; Low concentration, H; High concentration). (G) mRNA was extracted from HepG2 cells treated with the indicated concentrations of diclofenac for 24 h, and mRNA levels of GAPDH and PLIN2 were analyzed by qRT-PCR. (H) HepG2 cells were treated with diclofenac (300 μM) or oleate (100 μM), and the levels of PLIN2 were analyzed by western blotting. The densitometric quantification of PLIN2 is shown in the right panels. Data are presented as mean ± SD of at least 3 independent experiments, as analyzed by one-way ANOVA followed by Tukey's test. *p < 0.05, **p < 0.01, and ***p < 0.001, relative to the control group. #p < 0.05, relative to the oleate-treated group. n.s.; non-significant relative to the control group.

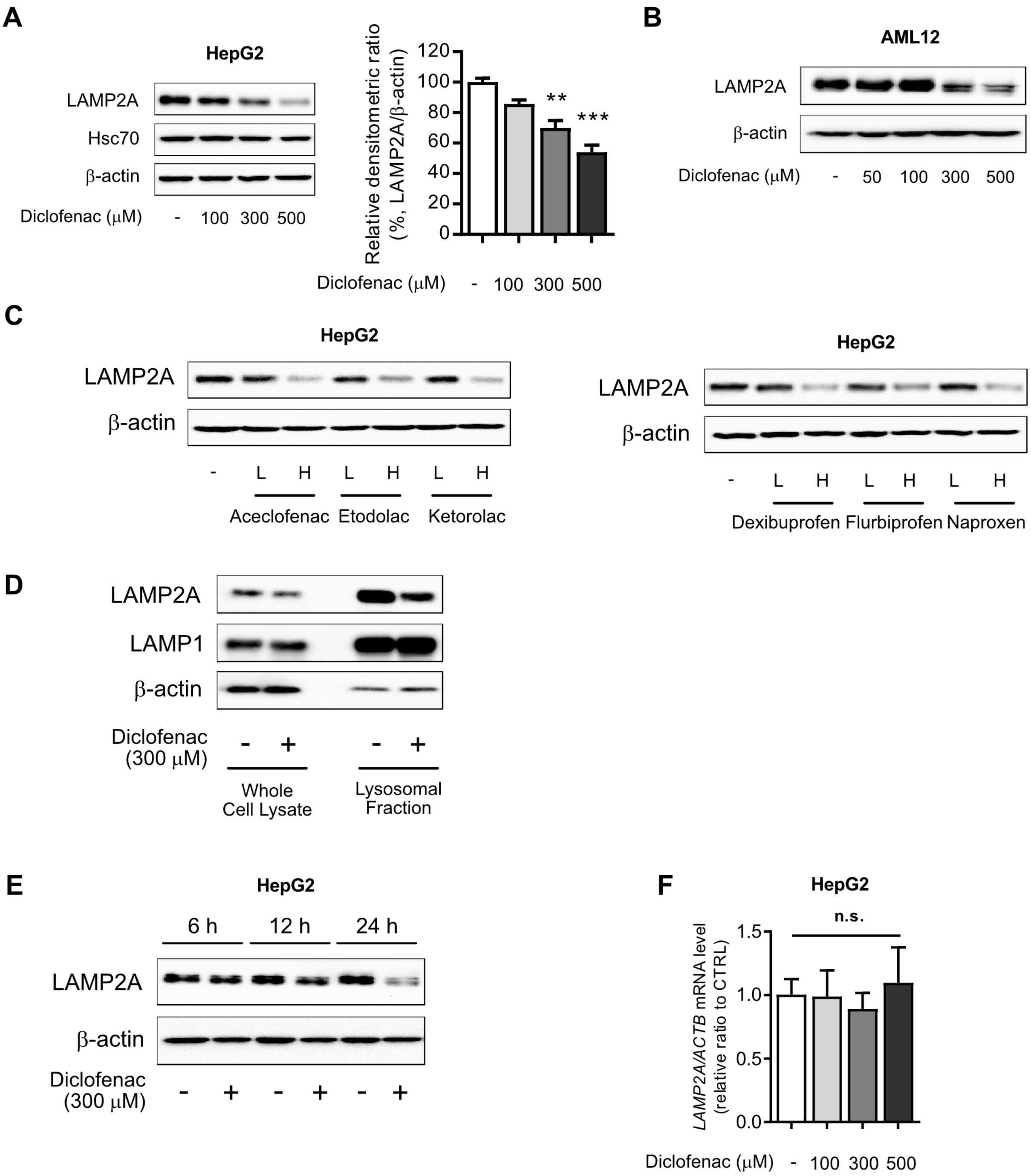
A
HepG2
LAMP2A
Hsc70
β-actin
Diclofenac (μM) - 100 300 500
Relative densitometric ratio (%, LAMP2A/β-actin)
120
100
80
60
40
20
0
**
***
Diclofenac (μM) - 100 300 500
B
AML12
LAMP2A
β-actin
Diclofenac (μM) - 50 100 300 500
C
HepG2
LAMP2A
β-actin
- L H L H L H
Aceclofenac Etodolac Ketorolac
HepG2
LAMP2A
β-actin
- L H L H L H
Dexibuprofen Flurbiprofen Naproxen
D
LAMP2A
LAMP1
β-actin
Diclofenac (300 μM) - + - +
Whole Cell Lysate
Lysosomal Fraction
E
HepG2
6 h 12 h 24 h
LAMP2A
β-actin
Diclofenac (300 μM) - + - + - +
F
HepG2
n.s.
LAMP2A/ACTB mRNA level (relative ratio to CTRL)
1.5
1.0
0.5
0.0
Diclofenac (μM) - 100 300 500

Supplementary Figure 2. The effect of diclofenac on downregulation of LAMP2A in murine normal hepatocyte cells (AML12) and in human hepatoma cells (HepG2).

(A) HepG2 cells were treated with the indicated concentration of several NSAIDs for 24 h, and the levels of LAMP2A were analyzed by western blotting. The right panels show the quantification of the LAMP2A level. (B) Western blot analysis of LAMP2A in AML12 cells treated with the indicated concentrations of diclofenac for 24 h. (C) Representative western blot images in Figure 3B. HepG2 cells were incubated with the indicated concentration of several NSAIDs for 24 h. The protein levels of LAMP2A were measured by western blot analysis. Representative western blot image are shown (L; Low concentration, H; High concentration). (D) HepG2 cells were treated with diclofenac (300 μM). 20 μg of proteins extracted from whole cell lysate or lysosomal fraction were subjected to western blot analysis. LAMP1 was as a lysosomal marker, and β-actin was a cytosolic marker. (E) Western blot analysis of LAMP2A in HepG2 cells treated with diclofenac (300 μM) at the indicated time point. (F) mRNA was extracted from HepG2 cells treated with the indicated concentration of diclofenac for 24 h, and mRNA levels of LAMP2A were analyzed by qRT-PCR. Data are presented as mean ± SD of at least 3 independent experiments, as analyzed by one-way ANOVA followed by Tukey's test. **$p < 0.01$, and ***$p < 0.001$, compared to the control group. n.s.; non-significant relative to the control group.

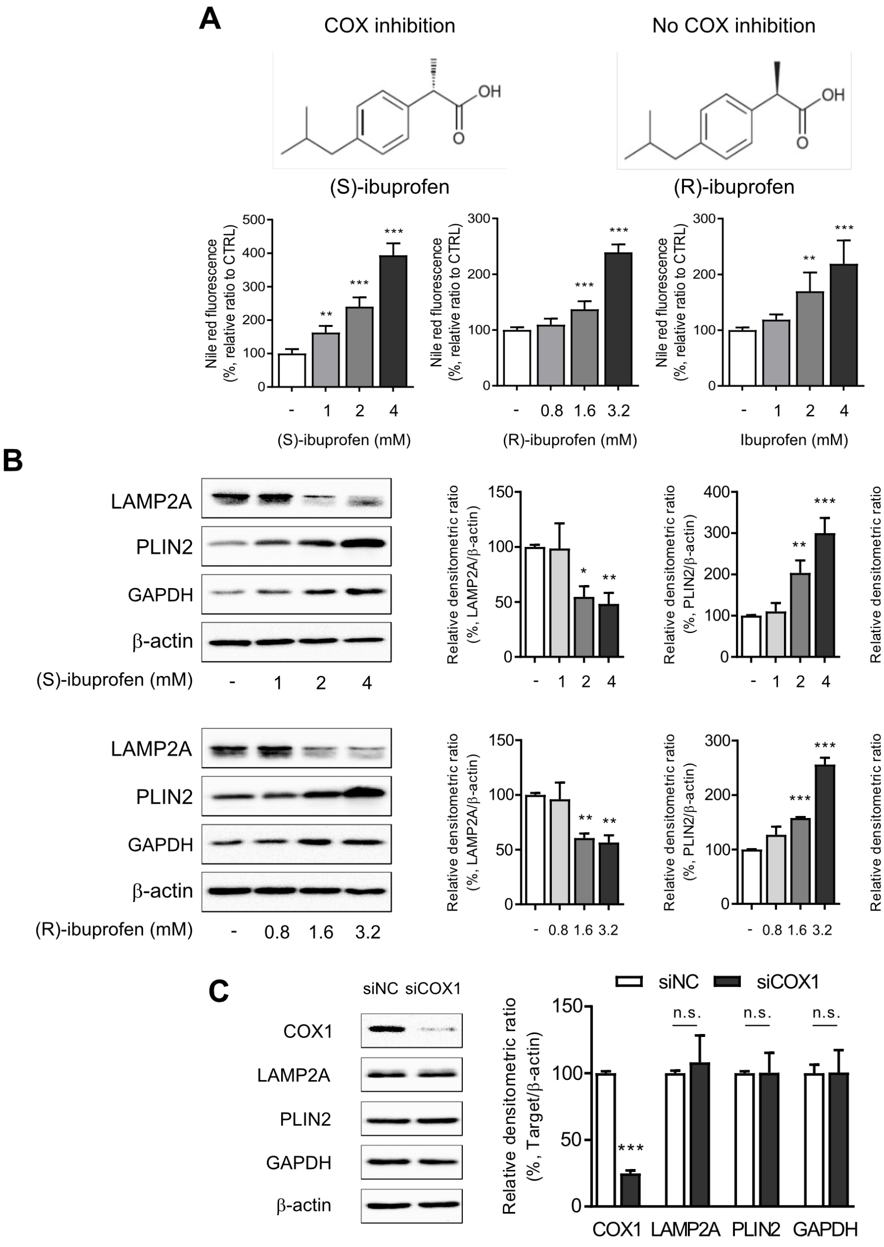

Supplementary Figure 3. CMA impairment by NSAIDs is independent of COX inhibition

(A) Chemical structure of (S)- and (R)- ibuprofen. HepG2 cells were incubated with (S)-ibuprofen or (R)-ibuprofen or racemic mixture of ibuprofen at the indicated concentration for 24 h. Intracellular lipid concentrations were quantified using Nile-red dye; excitation and emission wavelengths of 486 and 528 nm, respectively. (B) The protein levels of LAMP2A, PLIN2, and GAPDH were measured by western blot analysis. The right panels show the densitometric quantification of target proteins. (C) MPH were transfected with control or COX1 siRNA. The protein levels of COX1, COX2, LAMP2A, PLIN2, and GAPDH were measured by western blot analysis. Representative western blot images and the densitometric quantification of proteins are shown. Data are presented as mean ± SD of at least 3 independent experiments, as analyzed by one-way ANOVA followed by Tukey's test. $^{**}p < 0.01$, and $^{***}p < 0.001$, relative to the control group.

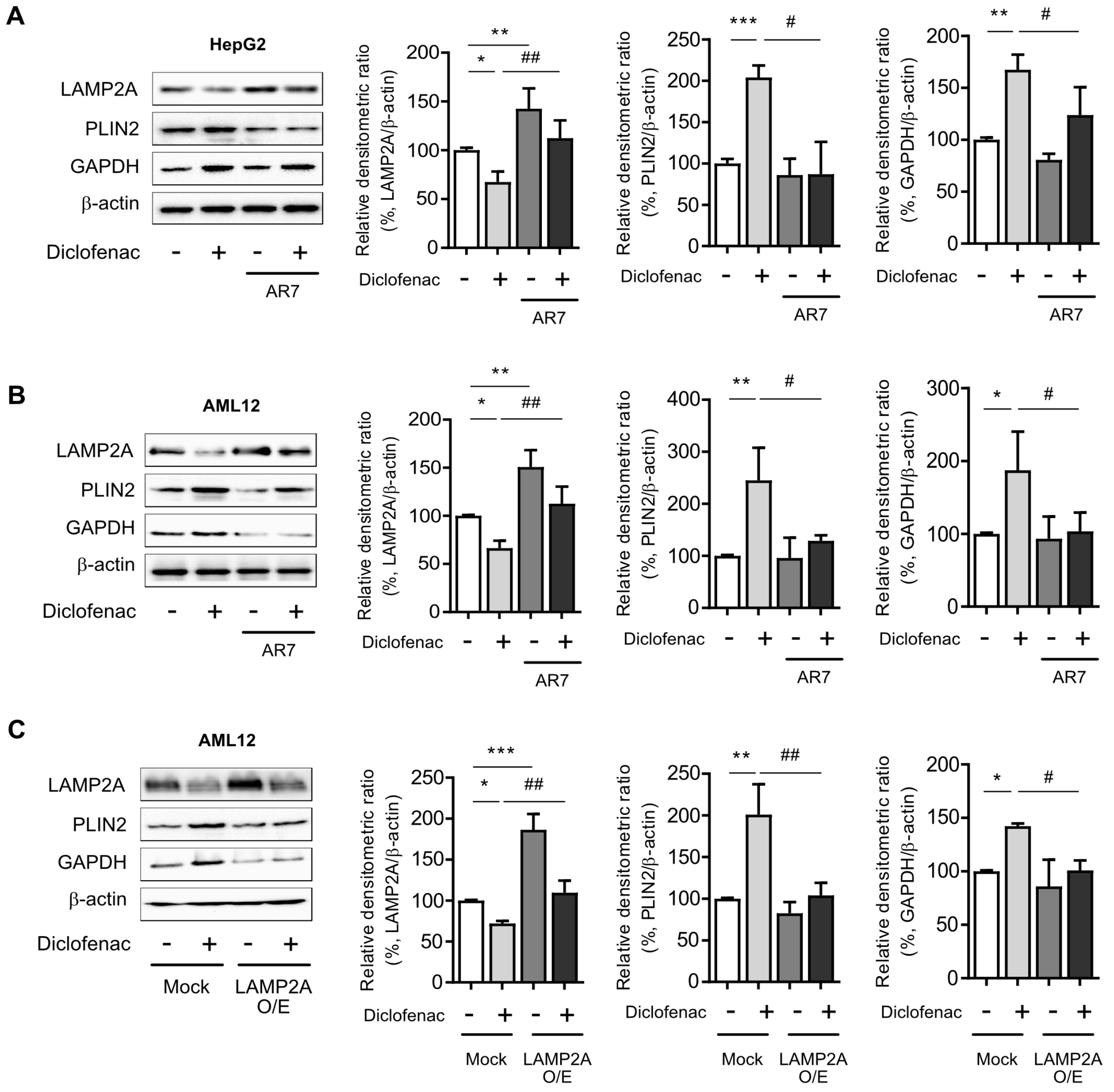

Supplementary Figure 4. Reactivation of chaperone-mediated autophagy restores diclofenac-induced inhibition of chaperone-mediated autophagy

(A) HepG2 cells were incubated with diclofenac (300 μM) or AR7 (10 μM) for 24 h. The protein levels of LAMP2A, PLIN2, and GAPDH were measured by western blot analysis. Representative western blot images and the densitometric quantification of proteins are shown. (B) AML12 cells were incubated with diclofenac (300 μM) or AR7 (10 μM) for 24 h. The protein levels of LAMP2A, PLIN2, GAPDH were measured by western blot analysis. (C) AML12 cells were transfected with pCMV-LAMP2A plasmid and were incubated with diclofenac for 24 h and subjected to western blot analysis with antibodies against LAMP2A, PLIN2 and GAPDH. Data are presented as mean ± SD of at least 3 independent experiments, as analyzed by one-way ANOVA followed by Tukey's test. *p < 0.05, **p < 0.01, ***p < 0.001, relative to the control group. #p < 0.05, ##p < 0.01, relative to diclofenac-treated group.

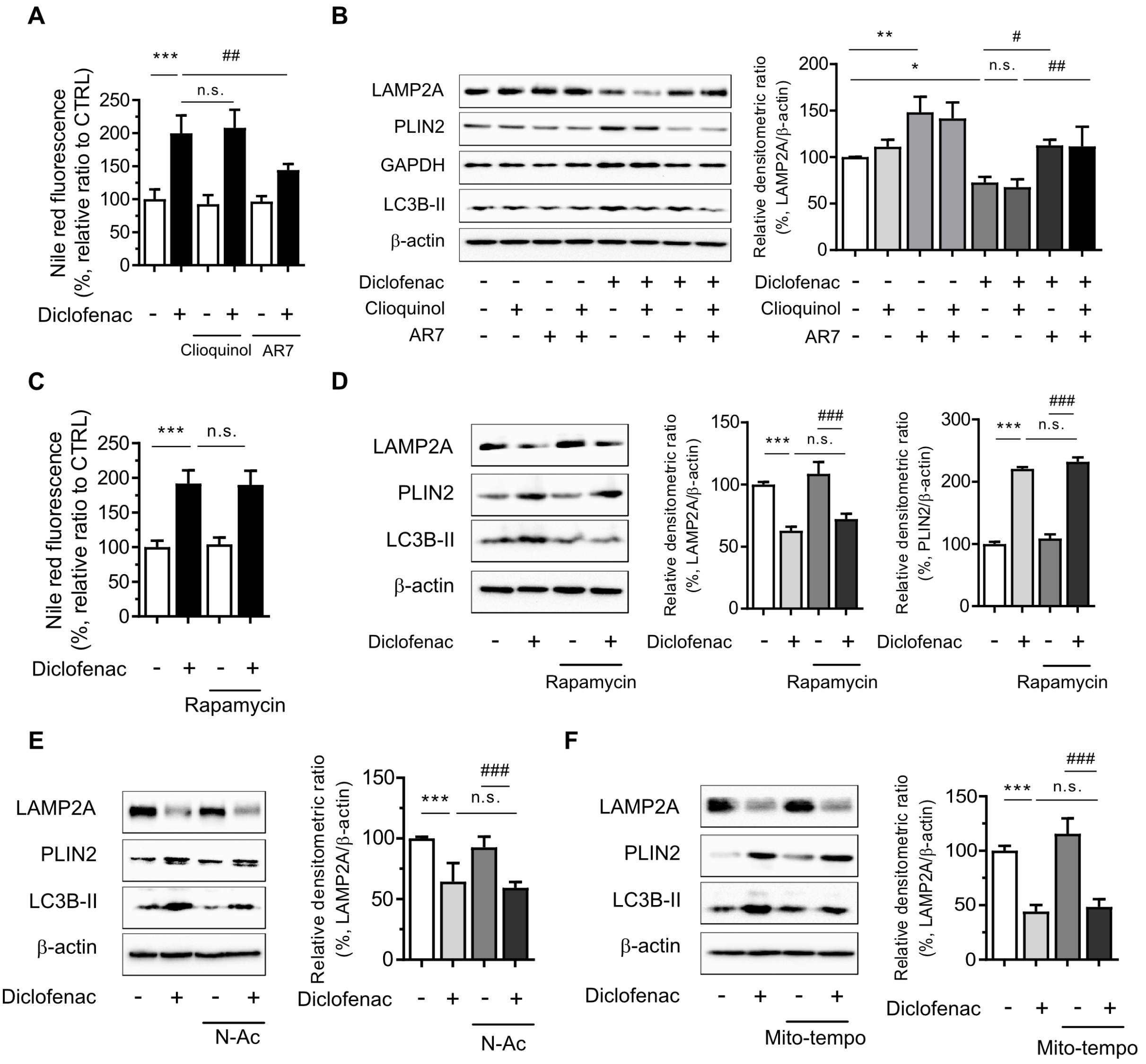

Supplementary Figure 5. CMA inhibition by diclofenac is independent of macroautophagy inhibition or oxidative stress

(A, B) MPH were incubated with diclofenac (300 μM) or Clioquinol (10 μM), AR7 (10 μM) for 24 h. (A) Intracellular lipid concentrations were quantified using Nile-red dye; excitation and emission wavelengths of 486 and 528 nm, respectively. (B) The protein levels of LAMP2A, PLIN2, GAPDH, and LC3B-II were measured by western blot analysis. Representative Western blot images and the relative quantification of LAMP2A are shown. (C, D) MPH were incubated with diclofenac (300 μM) or Rapamycin (0.5 μM) for 24 h. (C) Intracellular lipid concentrations were quantified using Nile-red dye; excitation and emission wavelengths of 486 and 528 nm, respectively. (D) The protein levels of LAMP2A, PLIN2, and LC3B-II were measured by western blot analysis. Representative western blot images and the densitometric quantification of proteins are shown. (E, F) MPH were pretreated with *N*-acetylcysteine (N-Ac; 5 mM) or Mito-Tempo (10 μM) for 1 h, and further incubated with diclofenac (300 μM; 24 h). The protein levels of LAMP2A, PLIN2, and LC3B-II were measured by

western blot analysis. Representative Western blot images and the relative quantification of LAMP2A are shown. Data are presented as mean ± SD of at least 3 independent experiments, as analyzed by one-way ANOVA followed by Tukey's test. *$p < 0.05$, **$p < 0.01$, ***$p < 0.001$, relative to the control group. #$p < 0.05$, ##$p < 0.01$, ###$p < 0.001$, relative to the indicated group. n.s.; non-significant relative to the indicated group.

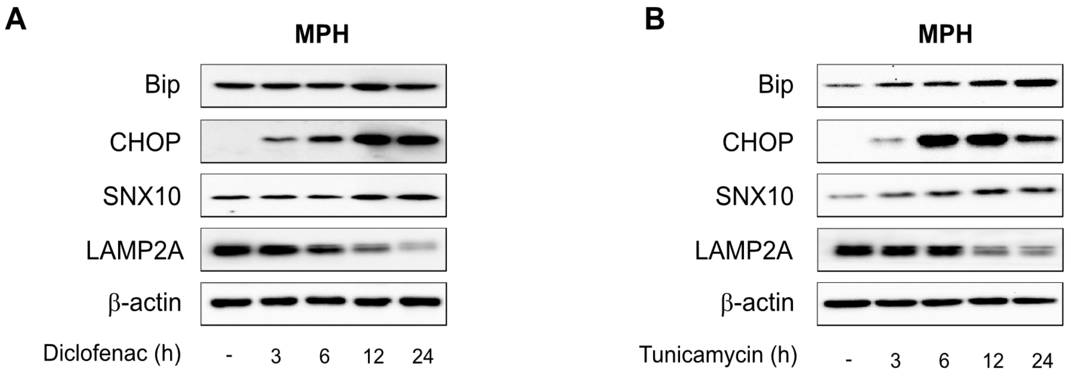

Supplementary Figure 6. Diclofenac or tunicamycin inhibits chaperone-mediated autophagy in MPH in a time-dependent manner

(A) MPH were treated with diclofenac (300 μM) as the indicated time point and subjected to western blot analysis with antibodies against Bip, CHOP, and SNX10. (B) MPH were treated with tunicamycin (3 μg/ml) at the indicated time point and the protein levels of Bip, CHOP, SNX10 and LAMP2A were measured by western subjected to western blot analysis.

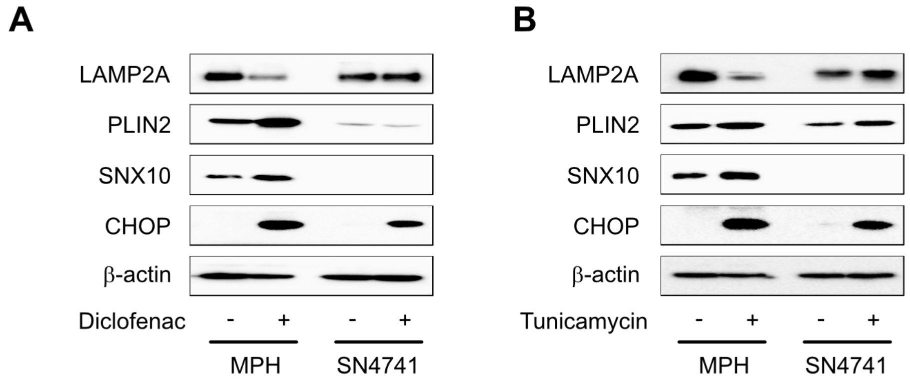

Supplementary Figure 7. The expression of SNX10 is deficient in SN4741 cells

(A) MPH and SN4741 cells were treated with diclofenac (300 μM) for 24 h and subjected to western blot analysis with antibodies against LAMP2A, PLIN2, SNX10 and CHOP. (B) MPH and SN4741 cells were treated with tunicamycin (3 μg/ml) for 12 h and subjected to western blot analysis with antibodies against LAMP2A, PLIN2, SNX10 and CHOP.

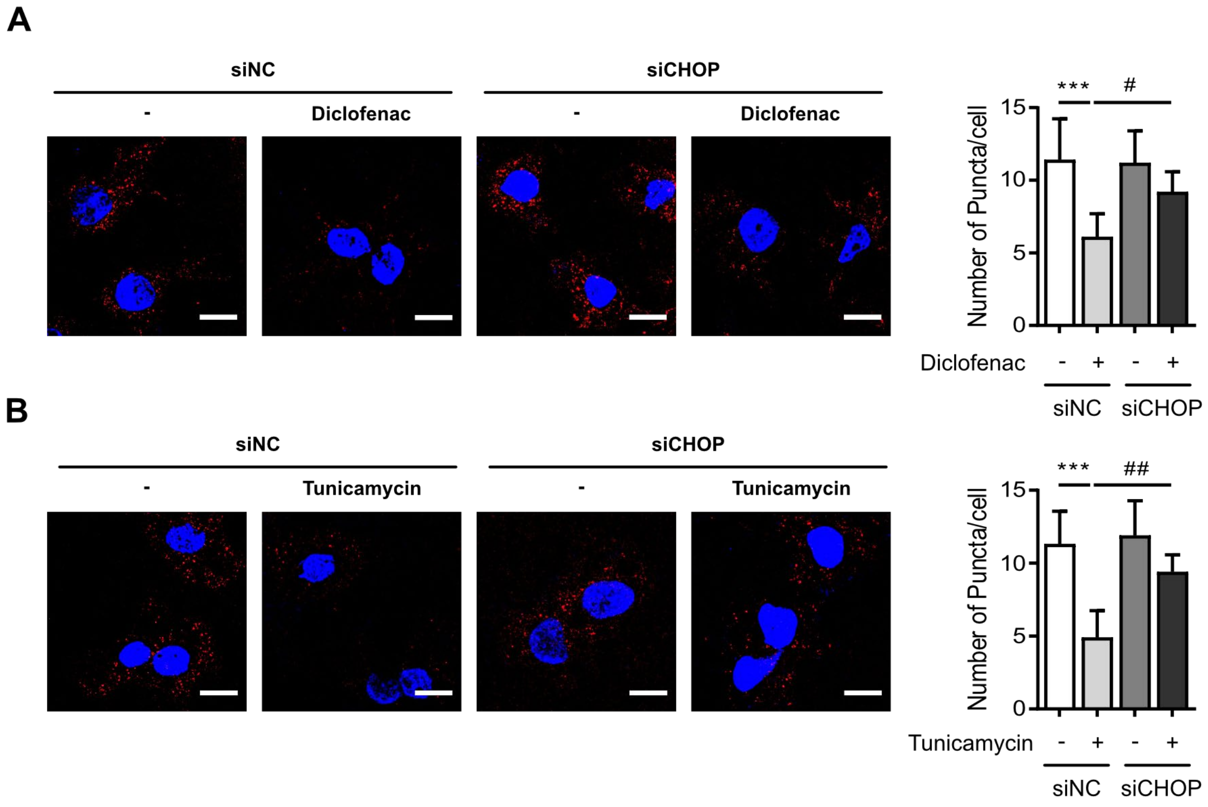

Supplementary Figure 8. The inhibition of CMA was rescued by knockdown of CHOP

(A, B) HepG2 cells were treated with diclofenac (300 μM) for 24 h or tunicamycin (3 μg/ml) for 12 h after transfection with control siRNA or each specific siRNA against CHOP followed by transfection with pSIN-PAmCherry-KFERQ-NE plasmid. Representative fluorescent images and quantification of PAmCherry-KFERQ puncta are shown (Red puncta; PAmCherry, Blue; DAPI). Data are presented as mean ± SD of 3 independent experiments, as analyzed by one-way ANOVA followed by Tukey's test. ***$p < 0.001$, relative to the control group. #$p < 0.05$, relative to the diclofenac-treated group. ##$p < 0.01$, relative to the tunicamycin-treated group.

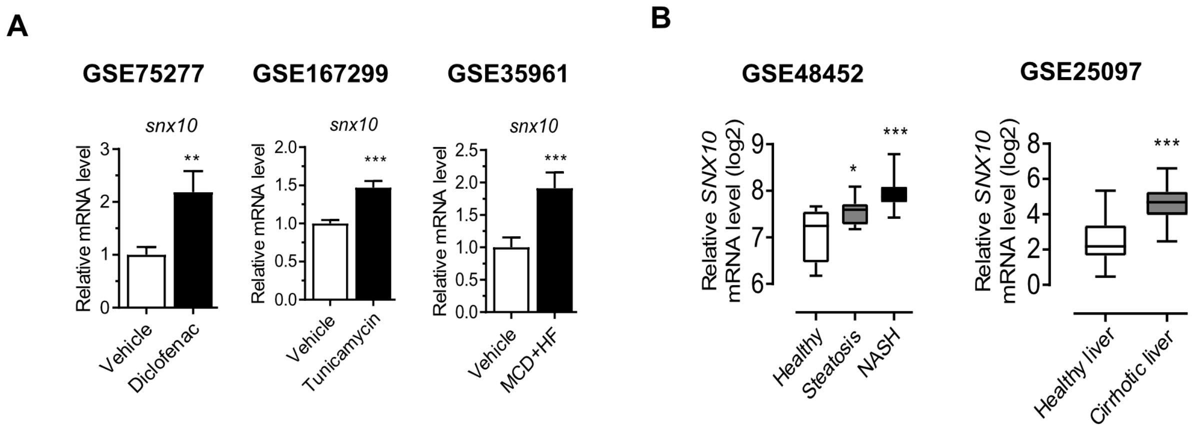

Supplementary Figure 9. SNX10 expression is increased in liver disease model.

(a) Microarray data from mouse diclofenac treated model or mouse tunicamycin treated model or mouse methionine-choline deficient (MCD) diet with high-fat (HF) were analyzed by the GEO integrated analysis tool GEO2R. (b) Microarray data from human simple steatosis, non-alcoholic steatohepatits or human liver cirrhosis were analyzed by the GEO integrated analysis tool GEO2R Data are presented as means ± SD. * $p < 0.05$, **$p < 0.01$, and ***$p < 0.001$, represent significant differences relative to the vehicle or health liver group using Student's t-test.

Supplementary Table 1. List of Primers for qRT-PCR

Species	Gene	Forward Primer (5′ to 3′)	Reverse Primer (5′ to 3′)
Human	*GAPDH*	AACGGATTTGGTCGTATT	GCTCCTGGAAGATGGTGA
Human	*PLIN2*	GTAGAGTGGAAAAGGAGCAT	CAGTCAGCTGAGGATAAAAGG
Human	*LAMP2A*	CTCTGCGGGGTCATGGTG	CGCACAGCTCCCAGGACT
Human	*SNX10*	TTGAGTCAGCAAACTCCGC	GGACACGATCAATCTGGGCG
Human	*ACTB*	AGGCACCAGGGCGTGAT	GCCCACATAGGAATCCTTCTGAC
Mouse	*Gapdh*	CGGTGCTGAGTATGTCGT	CTTCTGGGTGGCAGTGAT
Mouse	*Plin2*	CAGCCAACGTCCGAGATTG	CACATCCTTCGCCCCAGTT
Mouse	*Lamp2a*	AGGTGCTTTCT TGTCTAGAGCGT	AGAATAAGTAGTACTCCTCCCAGAGCTGC
Mouse	*Snx10*	GGGTTCGCTCTTAGGCGCTCG	GGACACGATCAACACGCGGTTCT
Mouse	*Actb*	CTGTCCCTGTATGCCTCTG	ATGTCACGCACGATTTCC
